# Validation of a two-fluid turbulence model in comsol multiphysics for the problem of flow around aerodynamic profiles

**DOI:** 10.1038/s41598-024-52673-5

**Published:** 2024-01-27

**Authors:** Z. M. Malikov, M. E. Madaliev, S. L. Chernyshev, A. A. Ionov

**Affiliations:** 1 Institute of Mechanics and Seismic Resistance of Structures of the Academy of Sciences of the Republic of Uzbekistan, Tashkent, Uzbekistan; 2 Fergana Polytechnic Institute, Fergana, Uzbekistan; 3 Central Aero-Hydrodynamic Institute Named After Professor N.E. Zhukovsky, Zhukovsky, Moscow Region Russia; 4 Moscow Institute of Physics and Technology (MIPT), Dolgoprudny, Moscow Region Russia

**Keywords:** Mathematics and computing, Physics

## Abstract

The article presents a study of a two-fluid turbulence model in the Comsol Multiphysics software package for the problem of a subsonic flow around the DSMA661 and NACA 4412 airfoils with angles of attack of 0 and 13.87 degrees, respectively. In this paper, the finite element method is used for the numerical implementation of the turbulence equations. To stabilize the discretized equations, stabilization by the Galerkin least squares method was used. The results obtained are compared with the results of other RANS, LES, DES models and experimental data. It is shown that in the case of continuous flow around the DSMA661 airfoil, the results of the two-fluid model are very close to the SST results and are in good agreement with the experimental data. When flowing around the NACA 4412 airfoil, flow separation occurs and a recirculation zone appears. It is shown that in such cases the two-fluid model gives more accurate results than other turbulence models. Implementation of the Comsol Multiphysics software package showed good convergence, stability, and high accuracy of the two-fluid turbulence model.

## Introduction

Computational Fluid Dynamics (CFD) plays a critical role in the aerospace industry as it allows us to optimize the aerodynamic characteristics of aircraft, space, and other flying machines. For example, it helps develop efficient airfoils, wings, and control surfaces to reduce drag and improve lift. CFD is used to study flow patterns and combustion processes in gas turbine engines and rocket propulsion systems. It helps to optimize engine design, improve fuel efficiency and reduce emissions. CFD is used to analyze and predict heat transfer phenomena such as conduction, convection, and radiation in aerospace systems. It is important for thermal management and to ensure the structural integrity of components exposed to high temperatures.

Overall, computational fluid dynamics has revolutionized the aerospace industry, allowing engineers to gain valuable insight into complex fluid flow phenomena and optimize designs before creating costly physical prototypes. CFD has significantly reduced development time and costs while improving the safety, efficiency, and productivity of aerospace systems.

One of the main driving forces behind the growth of computational fluid dynamics was the aerospace industry. Over the past 40 years, it has evolved from a useful method of analysis to a mainstream design tool. In companies like Boeing, much of the early wing design work is done almost exclusively using CFD^1,2^.

It is known that turbulence is a problem of classical physics to be solved The importance of this problem lies in the fact that the vast majority of flows occurring in nature and in various technological processes are precisely of a turbulent nature. To date, there are several approaches to the mathematical modeling of turbulence. The most common is the Reynolds approach. Based on this approach, a Reynolds-averaged Navier–Stokes system of equations (RANS) is obtained. However, as is known, this system of equations is not closed. To close the resulting system of equations, a large number of different mathematical models were proposed. These models are based on the hypotheses of Boussinesq^3^, Kolmogorov^4^, Prandtl^5^, Karman^6^, etc. The NASA turbulence database^7^ provides a comparative analysis of various semi-empirical models. From this analysis, we can conclude that the models of Spalart and Allmaras^8^, and Menter k − ω SST^9–11^ have the highest ratings. To date, these models have been used to obtain numerical solutions to many important practical problems^12–14^.

An important step in the development of computational methods is the verification of the created mathematical models in wind tunnels by correcting the data obtained, excluding boundary induction^15–18^

However, at present, despite the fact that RANS methods are widely used, there are hydrodynamic problems the solution to which cannot give satisfactory results. These include the problem of transition from laminar to turbulent regime and separated flows.

Recently, due to the rapid development of computer technology, direct methods of turbulence simulation (DNS, LES) have become increasingly popular. These methods have high accuracy, but require large computational resources. Therefore, it will take some time to use them in solving engineering problems. The so-called hybrid RANS/LES methods, called the methods of detached-eddy simulation (DES) of vortices^14^, received good development. The essence of this method is that near solid surfaces, where high resolution of computational cells is required, the RANS model is used, and far from the walls, the LES model is used. The approach significantly saves computational resources and gives high-accuracy results^19^.

Recently, the two-fluid model of turbulence has become increasingly popular^20,21^. This turbulence model is based on the dynamics of two fluids, which, unlike the Reynolds approach, leads to a closed system of equations. These articles show that the two-fluid model is a low-Reynolds one and capable of describing complex anisotropic turbulent flows. In^22^, a two-fluid turbulence model was used to solve the problem of the transverse flow around a square cylinder. Comparison with experimental data showed high accuracy of the model.

Up to now, the numerical implementation of the two-fluid turbulence model has been conducted using proprietary computational codes. However, the model becomes more important if it is implemented using well-known software packages. To date, special programs such as ANSYS Fluent, Solidworks, Comsol Multiphysics, etc. can be used to simulate an airfoil^23–26^.

ANSYS Fluent is a widely used computational fluid dynamics (CFD) software package developed by ANSYS Inc. It is a powerful tool for modeling and analyzing fluid flow, heat transfer and related phenomena. ANSYS Fluent uses the control volume method to solve fluid dynamics equations.

COMSOL Multiphysics is a powerful software package for modeling physical phenomena in various disciplines, including fluid dynamics, heat transfer, structural mechanics, electromagnetism, and chemical reactions. COMSOL Multiphysics uses the Finite Element Method to solve hydrodynamic equations. COMSOL Multiphysics offers several advantages over ANSYS Fluent and Solidworks, particularly in terms of versatility, multiphysics capabilities, and customization options. In COMSOL Multiphysics, the users have the option to define and solve their own partial differential equations (PDEs) using the Custom PDE functions. This feature enables the users to model and simulate specific physical phenomena that may not be addressed by the pre-built physics modules.

An attempt is done in this article to solve this problem and the following goals are posed:

1. Using the Custom PDE functions to implement a two-fluid turbulence model in the COMSOL Multiphysics software package.
2. Validation of the two-fluid turbulence model and verification of the computational algorithm on a number of simple test problems, such as flows around a flat plate with a zero pressure gradient, a DSMA661 airfoil with an angle of attack of 0 degrees and a NACA 4412 airfoil with an angle of attack of 13.87 degrees.
3. Compare the obtained results with the results of the well-known SST turbulence model (built into the COMSOL Multiphysics program) and experimental data from the NASA Turbulence Modeling Resource (TMR) website^7^.

## Two-fluid turbulence model.

The description of this model is presented in several publications by one of the authors of this article^20–22^. The main equations for studying the tasks posed are the hydrodynamic equations of the two-fluid model^24^ for an incompressible medium

1 $$ \left\{ \begin{gathered} \frac{{\partial V_{j} }}{{\partial x_{j} }} = 0, \hfill \\ \frac{{\partial V_{i} }}{\partial t} + \frac{{\partial V_{j} V_{i} }}{{\partial x_{j} }} + \frac{\partial p}{{\rho \partial x_{i} }} = \frac{\partial }{{\partial x_{j} }}\left[ {\nu (\frac{{\partial V_{i} }}{{\partial x_{j} }} + \frac{{\partial V_{j} }}{{\partial x_{i} }}) - \vartheta_{j} \vartheta_{i} )} \right], \hfill \\ \frac{{\partial \vartheta_{i} }}{\partial t} + \frac{{\partial V_{j} \vartheta_{i} }}{{\partial x_{j} }} = - \vartheta_{j} \frac{{\partial V_{i} }}{{\partial x_{j} }} + \frac{\partial }{{\partial x_{j} }}\left[ {\tilde{\nu }_{ji} (\frac{{\partial V_{i} }}{{\partial x_{j} }} + \frac{{\partial V_{j} }}{{\partial x_{i} }})} \right] + F_{si} + F_{fi} , \hfill \\ \tilde{\nu }_{ji} = 3\nu + 2\left| {\frac{{\vartheta_{i} \vartheta_{j} }}{{def(\vec{V})}}} \right|\;\;\;i \ne j,\;\;\;\tilde{\nu }_{ii} = 3\nu + \frac{{\sum\limits_{k = 1}^{3} {\vartheta_{k} \vartheta_{k} \left| {\frac{{\partial \vartheta_{k} }}{{\partial x_{k} }}} \right|} }}{{def(\vec{V})\sum\limits_{k = 1}^{3} {\left| {\frac{{\partial \vartheta_{k} }}{{\partial x_{k} }}} \right|} }}, \hfill \\ \vec{F}_{f} = - K_{f} \vec{\vartheta },\;\;\;\vec{F}_{s} = C_{s} rot\vec{V} \times \vec{\vartheta }. \hfill \\ \end{gathered} \right. $$

In the given system of equations $V_{i}$ is the component of the average flow velocity, $\vartheta_{i}$ is the component of the relative velocity, $\tilde{\nu }_{ji}$ is the molar viscosity tensor, $p$ is the pressure, $\rho$ is the density of the medium, $\nu$ is the molecular viscosity, *K*_*f*_ is the friction coefficient, $C_{s}$ is the coefficient at the Saffman force, ${\text{def}}(\vec{V})$ is the strain rate, determined as:

2 $$ {\text{def}}(\vec{V}) = \sqrt {2S_{ij} S_{ij} } ,\;\;\;S_{ij} = \frac{1}{2}\left( {\frac{{\partial V_{i} }}{{\partial x_{j} }} + \frac{{\partial V_{j} }}{{\partial x_{i} }}} \right). $$

The coefficient of friction is found from the following relation:

3 $$ \,\,K_{f} = C_{1} \lambda_{\max } + C_{2} \frac{{\left| {d \cdot \vartheta } \right|}}{{d^{2} }}. $$

In this expression, *d* is the nearest distance to the solid wall, *λ*_max_ is the real part of the largest root of the characteristic equation $\det (A - \lambda E) = 0,\,$ where *A* is the matrix

4 $$ \begin{aligned} & A = \left| {\begin{array}{*{20}c} { - \frac{{\partial V_{1} }}{{\partial x_{1} }}} & { - \frac{{\partial V_{1} }}{{\partial x_{2} }} - C_{s} \zeta_{3} } & { - \frac{{\partial V_{1} }}{{\partial x_{2} }} + C_{s} \zeta_{2} } \\ { - \frac{{\partial V_{2} }}{{\partial x_{1} }} + C_{s} \zeta_{3} } & { - \frac{{\partial V_{2} }}{{\partial x_{2} }}} & { - \frac{{\partial V_{2} }}{{\partial x_{3} }} - C_{s} \zeta_{1} } \\ { - \frac{{\partial V_{3} }}{{\partial x_{1} }} + C_{s} \zeta_{2} } & { - \frac{{\partial V_{3} }}{{\partial x_{2} }} + C_{s} \zeta_{1} } & { - \frac{{\partial V_{3} }}{{\partial x_{3} }}} \\ \end{array} } \right|, \\ & \zeta = rot\overrightarrow {V} . \\ \end{aligned} $$

Constant patterns are $C_{s} = 0.2,\;C_{1} = 0.7825,\;C_{2} = 0.306.$

Consider a two-dimensional stationary solution to system (1). For the finite element method, the application of the standard Galerkin method will lead to a weak form:

5 $$ \left\{ \begin{gathered} \nu \int\limits_{\Omega } {\nabla {\mathbf{u}}:\nabla {\mathbf{v}}} + \int\limits_{\Omega } {\left( {{\mathbf{u}} \cdot \nabla {\mathbf{u}}} \right) \cdot {\mathbf{v}}} - \int\limits_{\Omega } {p\left( {\nabla \cdot {\mathbf{v}}} \right)} = \int\limits_{\Omega } {{\mathbf{f}} \cdot {\mathbf{v}}} , \hfill \\ \tilde{\nu }\int\limits_{\Omega } {\nabla {\tilde{\mathbf{u}}}:\nabla {\tilde{\mathbf{v}}}} + \int\limits_{\Omega } {\left( {{\mathbf{u}} \cdot \nabla {\tilde{\mathbf{u}}}} \right) \cdot {\tilde{\mathbf{v}}}} = \int\limits_{\Omega } {{\mathbf{f}} \cdot {\tilde{\mathbf{v}}}} , \hfill \\ \int\limits_{\Omega } {q\left( {\nabla \cdot {\mathbf{u}}} \right)} = 0. \hfill \\ \end{gathered} \right. $$

Here, the equations of system (5) are a weak form of the equation of motion for the averaged and relative velocities, as well as the equation of continuity. Here, ${\mathbf{v}}$, ${\tilde{\mathbf{v}}}$, and ${\mathbf{q}}$ are weight functions for the average velocity $V$, relative velocity $\vartheta$, and pressure $p$, respectively.

Using the Finite Element Method (FEM) as a discretization method for finding solutions to turbulence models can be quite a challenge. The standard Galerkin problem statement deals with potential sources of numerical instabilities. This is, for example, the case when convection or reaction conditions prevail in the flow^27,28^. It is difficult to find such a stabilization that makes the solution of equations as reliable as possible. A stabilized FEM formulation can be created by adding grid-dependent, consistent, and numerically stabilizing terms to the standard Galerkin method. Various stabilization methods were proposed, many of which are based on the Petrov–Galerkin (PG) approach, which uses a modification of the standard Galerkin weight term. Examples of popular SG methods are the Streamline-Upwind/Petrov–Galerkin scheme^29–33^ (SUPG) and the pressure stabilization scheme/Petrov–Galerkin^34^ (SUPG). Over the years, the Petrov–Galerkin method has been developed and gave rise to new, more advanced methods. One of them is the Galerkin Least Squares (GLS) stabilization method. GLS is a general stabilization method applicable to a wide range of problems^35–39^. Its theoretical basis is that the test function should be chosen so as to minimize the squared residual of the equations. By working with matrices and not just with scalar equations, GLS can be formulated for systems of transport equations.

By adding the least squares term, we get the Galerkin/least squares formula for the two-fluid equations as shown below:

6 $$ \left\{ \begin{gathered} \nu \int\limits_{\Omega } {\nabla {\mathbf{u}}:\nabla {\mathbf{v}}} + \int\limits_{\Omega } {\left( {{\mathbf{u}} \cdot \nabla {\mathbf{u}}} \right) \cdot {\mathbf{v}}} - \int\limits_{\Omega } {p\left( {\nabla \cdot {\mathbf{v}}} \right)} + R_{GLS} = \int\limits_{\Omega } {{\mathbf{f}} \cdot {\mathbf{v}}} , \hfill \\ \tilde{\nu }\int\limits_{\Omega } {\nabla {\tilde{\mathbf{u}}}:\nabla {\tilde{\mathbf{v}}}} + \int\limits_{\Omega } {\left( {{\mathbf{u}} \cdot \nabla {\tilde{\mathbf{u}}}} \right) \cdot {\tilde{\mathbf{v}}}} + \tilde{R}_{GLS} = \int\limits_{\Omega } {{\mathbf{f}} \cdot {\tilde{\mathbf{v}}}} , \hfill \\ \int\limits_{\Omega } {q\left( {\nabla \cdot {\mathbf{u}}} \right)} = 0. \hfill \\ \end{gathered} \right. $$

where $R_{GLS}$ and $\tilde{R}_{GLS}$ are given as:

7 $$ \left\{ \begin{gathered} R_{GLS} = \tau \left( {{\mathbf{u}} \cdot \nabla {\mathbf{v}} - \nu \nabla^{2} {\mathbf{v}}} \right)\left( {{\mathbf{u}} \cdot \nabla {\mathbf{u}} - \nu \nabla^{2} {\mathbf{u}} - {\mathbf{f}} \cdot {\mathbf{v}}} \right) + \tau_{p} \left( {\nabla q\nabla p} \right), \hfill \\ \tilde{R}_{GLS} = \tilde{\tau }\left( {{\mathbf{u}} \cdot \nabla {\tilde{\mathbf{v}}} - \tilde{\nu }\nabla^{2} {\tilde{\mathbf{v}}}} \right)\left( {{\mathbf{u}} \cdot \nabla {\tilde{\mathbf{u}}} - \tilde{\nu }\nabla^{2} {\tilde{\mathbf{u}}} - {\mathbf{f}} \cdot {\tilde{\mathbf{v}}}} \right). \hfill \\ \end{gathered} \right. $$

Parameters $\tau$ and $\tilde{\tau }$ for stationary problems are determined by the following expression:

8 $$ \left\{ \begin{gathered} \tau = \min \left( {\frac{h}{{2\rho \left| {\mathbf{u}} \right|}},\frac{{h^{2} }}{48\nu }} \right), \hfill \\ \tilde{\tau } = \min \left( {\frac{h}{{2\rho \left| {\mathbf{u}} \right|}},\frac{{h^{2} }}{{48\;\min \left( {\tilde{\nu }_{11} ,\tilde{\nu }_{12} } \right)}}} \right). \hfill \\ \end{gathered} \right. $$

And for pressure, parameter $\tau_{p}$ is determined by the following expression:

9 $$ \tau_{p} = \frac{{\left| {\mathbf{u}} \right|h}}{2}\min \left( {1,{\text{Re}}^{h} } \right). $$

where ${\text{Re}}^{h}$ is the Reynolds number of the element:

10 $$ {\text{Re}}^{h} = \frac{{\left| {\mathbf{u}} \right|h}}{\nu }. $$

## Solution method and boundary conditions

COMSOL Multiphysics offers a range of solvers to solve various types of problems in physics. The choice of solver depends on the type of physics being modeled, the complexity of the problem, the sought-for accuracy, and the available computational resources. To solve the equations of the two-fluid turbulence model, a fully coupled approach was used with the direct solver algorithm (PARDISO). Newton's iterative method with a damping factor of 0.1 was used. The iterative process for the problem of flow around a flat plate with zero pressure gradient lasted up to 250 iterations, and for the remaining problems, the iterative process continued up to 350 iterations. The tolerance factor is 1, the residual factor is 1000.

The standard SST turbulence model uses standard COMSOL Multiphysics solvers. The following boundary conditions were set for the SST model:

11 $$ \omega_{\infty } = 10\frac{{U_{\infty } }}{L},\;\;\;k_{\infty } = 0.1\frac{{\nu_{\infty } U_{\infty } }}{L}. $$

where L is the characteristic size of the streamlined body. The remaining boundary conditions were specified in the standard way.

For the problem of an aerodynamic profile, the initial distribution of velocity and pressure was given by the potential field of velocities. Assuming an irrotational inviscid flow, $\varphi$ the velocity potential is defined as

12 $$ {\mathbf{u}} = - \nabla \varphi . $$

The velocity potential must satisfy the continuity equation for an incompressible flow $\nabla {\mathbf{u}} = 0$. The continuity equation can be represented as the Laplace equation

13 $$ \nabla \left( { - \nabla \varphi } \right) = 0. $$

which is the potential flow equation.

After calculating the velocity potential, the pressure can be approximated using the Bernoulli equation for stationary flows:

14 $$ p = - \frac{\rho }{2}\left| {\nabla \varphi } \right|^{2} . $$

## Flat plate with zero pressure gradient

**Figure 1:**
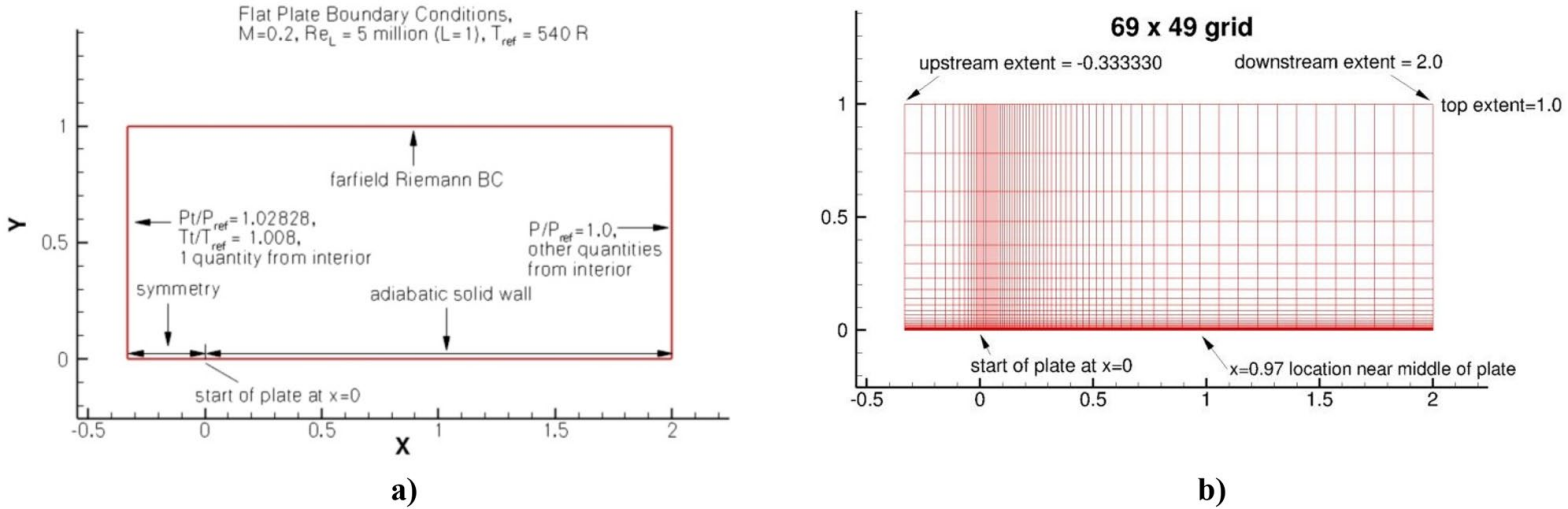
Flat plate with zero pressure gradient (**a**) boundary conditions and (**b**) computational grid and domain.

The main purpose of this experiment is to test the implementation of the two-fluid turbulence model in Comsol Multiphysics and compare the obtained results with the experimental data presented on the NASA website^7^. In the calculation, the plate length was L = 2 m with Reynolds number Re = 5,000,000 per unit length. In this case, the maximum thickness of the boundary layer is approximately 0.03 L, so the computational grid height was removed by distance y = L, which is sufficient to have little effect on the results. The turbulence intensity of the oncoming flow was 0.039%. The boundary conditions are shown in Fig. 1a, and an illustration of the computational grid and domain are shown in Fig. 1b. A computational grid of 69 × 49 in size, presented on the NASA website was used^7^.

**Figure 2:**
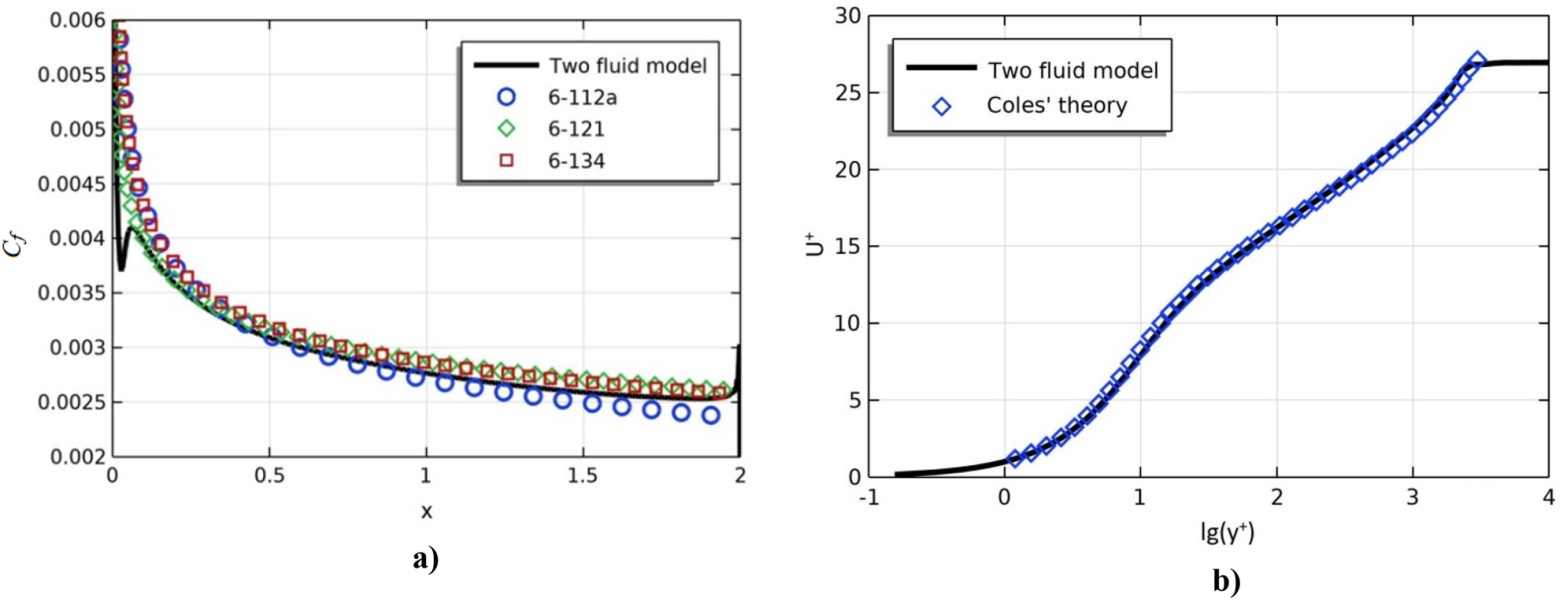
Dependence of the coefficient of friction along the plate (**a**), profile of the dimensionless longitudinal flow velocity on the dimensionless distance to the plate (**b**).

Below are comparisons of the obtained numerical results with known experimental data. Figure 2 shows: a) the dependence of the friction coefficient along the plate; b) the dimensionless longitudinal flow velocity as a function of the dimensionless distance to the plate, as well as the results of Cole’s theorie^40,41^.

Here $C_{f}$ is the coefficient of friction of the plate:

15 $$ C_{f} = \frac{2}{{\text{Re}}}\left( {\frac{{\partial V_{x} }}{\partial y}} \right). $$

**Figure 3:**
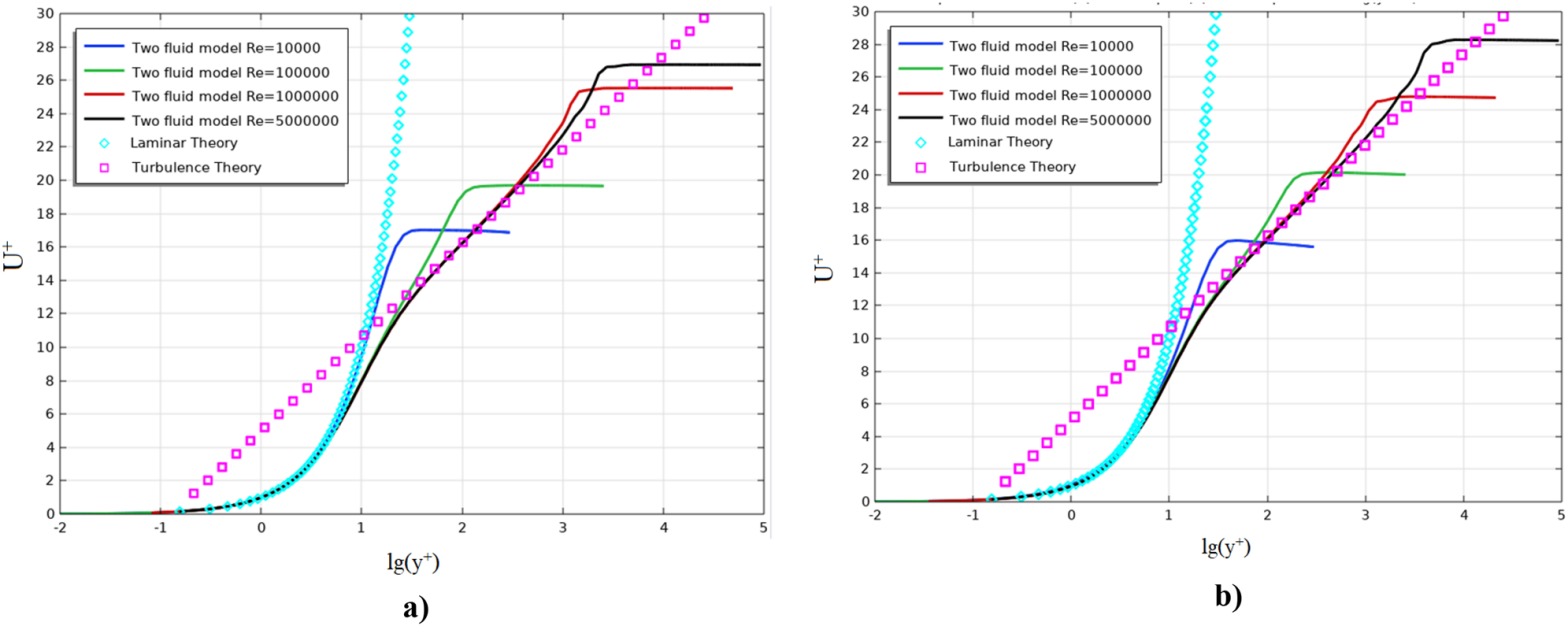
Dimensionless longitudinal flow velocity at various Reynolds numbers.

Figure 3 shows the profiles of the dimensionless longitudinal velocity at different Reynolds numbers in two sections: (a) x = 0.97 m and (b) x = 1.97 m.

The solid line in Fig. 3 shows the results of numerical calculation for the dimensionless longitudinal flow velocity depending on the dimensionless distance to the plate. Dimensionless velocities and distance were determined by the following formulas:

16 $$ u^{ + } = \frac{{V_{x} }}{{u^{*} }},\;\;\;y^{ + } = {\text{Re}} yu^{*} ,\;\;\;u^{*} = \sqrt {0.5C_{f} } . $$

Figures 2 and 3 show that, for all Reynolds numbers, the model well describes both laminar and turbulent zones^22^.

## Airfoil DSMA661

The DSMA661 airfoil is designed for low Reynolds flow. It was developed by the Delft University of Technology in the Netherlands. The DSMA 661 airfoil is commonly used in unmanned aerial vehicles (UAVs), especially those operating at low speeds. This airfoil has a relatively large maximum thickness of 16% of the chord length. It has moderate camber and is designed to provide good lift and low drag at low Reynolds numbers. The DSMA 661 airfoil is known for its stable and predictable behavior, making it suitable for a variety of UAV missions.

**Figure 4:**
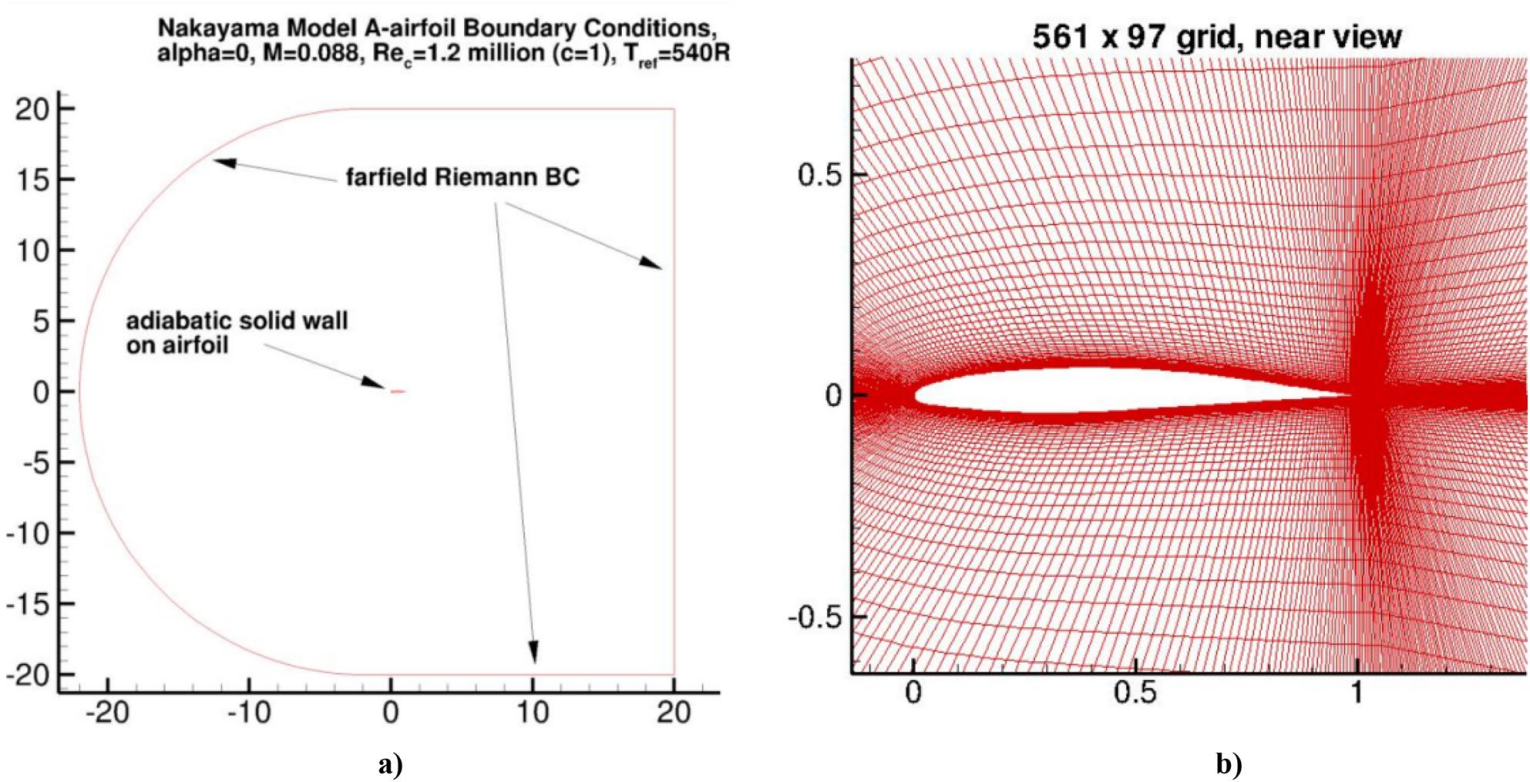
Airfoil DSMA661 (Model A). (**a**) boundary conditions; and (**b**) computational grid and area.

The DSMA661 airfoil^42^ provides another opportunity to evaluate the implementation of the proposed two-fluid model in Comsol Multiphysics. Four different two-dimensional grids were used in the work. Each coarser grid represents exactly every second point of the next finer grid, ranging from the 1121 × 193 fine grid to the coarsest 141 × 25 grid, presented on the NASA website^7^. There are 305 points on the fine grid along the trace from the trailing edge of the profile to the outflow boundary (39 points on the coarsest grid). The main results were taken for a grid of 561 × 97, and the remaining computational grids were used to check the grid convergence. The boundary conditions are shown in Fig. 4a, and an illustration of the computational grid and computational domain is shown in Fig. 4b.

For the problem at hand, the results of the well-known SST turbulence model are also obtained. The results are obtained with a free stream flow velocity of 18 m/s at zero angle of attack, which corresponds to a Reynolds number based on a chord of 1.2 million. For both models, the free flow turbulence intensity was 0.088%.

The distribution of the surface pressure coefficient on an airfoil is characterized by a change in pressure on its surface depending on the distance from a certain point. Typically, the analysis uses the surface pressure coefficient Cp, defined as the ratio of the pressure difference between a point on the surface of the profile and the pressure of the free flow to the dynamic pressure of the free flow.

17 $$ C_{p} = \frac{{p - p_{\infty } }}{{0.5\rho U_{0}^{2} }} $$

where p is the pressure at a point on the profile surface, P_∞_ is the pressure of the free flow, ρ is the density of the free flow, U_0_ is the velocity of the free flow.

**Figure 5:**
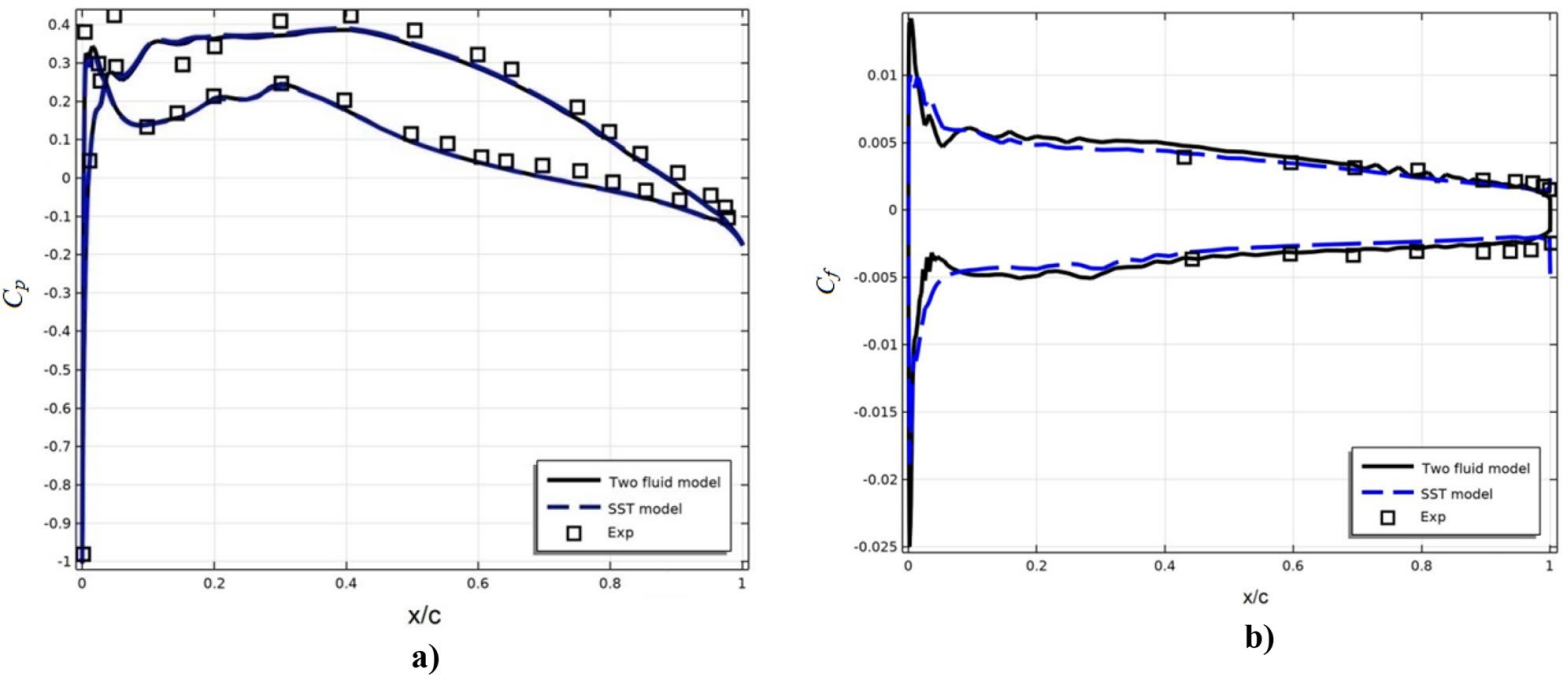
Research results (**a**) pressure coefficient (**b**) friction coefficient.

The distribution of the surface pressure coefficient on an airfoil can be used to analyze its aerodynamic characteristics such as lift, drag coefficient, etc. Figure 5a shows the distribution of the surface pressure coefficient C_p_ of the DSMA661 profile at an angle of attack $\alpha = 0^{0}$.

The distribution of the coefficient of skin friction on an airfoil is characterized by a change in the friction force on its surface depending on the distance from a certain point. The coefficient of skin friction *C*_*f*_ is defined as the ratio of the friction force acting on the surface of the profile to the dynamic pressure of the free flow.

18 $$ C_{f} = \frac{F}{{0.5\rho U_{0}^{2} S}} $$

where F is the friction force acting on the profile surface, S is the profile surface area oriented along the flow. Figure 5b illustrates the numerical and experimental results for *C*_*f*_ for both the top and bottom profile surfaces.

Figures 5 show that the results of both turbulence models practically coincide and are in good agreement with the experimental data.

Table 1 presents the errors in the deviation of numerical results from experimental data for *C*_*f*_ and *C*_*p*_.

**Table 1:** Advances in LES of Complex Flows.

*C*_*f*_ upper wall							
x/c	0.431521	0.5956	0.6963	0.7932	0.895	0.947	0.972
Exp	0.00388	0.00351	0.00314	0.00296	0.002167	0.0021	0.00198
Two fluid model	0.004737	0.00388	0.00333	0.00259	0.001984	0.0017	0.00167
δ %	0.0857	0.037	0.019	0.037	0.0183	0.04	0.031
SST model	0.004187	0.003514	0.002963	0.002351	0.001861	0.0016	0.00149
δ %	0.0307	0.00039	0.0177	0.0609	0.0306	0.05	0.049

*C*_*f*_ lower wall							
x/c	0.4431	0.5956	0.6937	0.7932	0.8966	0.9392	0.971576
Exp	− 0.0037	− 0.0032	− 0.0034	− 0.00316	− 0.00312	− 0.00309	− 0.00303
Two fluid model	− 0.0035	− 0.00309	− 0.00297	− 0.00284	− 0.0026	− 0.00258	− 0.00236
δ %	0.02	0.011	0.043	0.0315	0.0515	0.0508	0.0673
SST model	− 0.00315	− 0.00273	− 0.00248	− 0.00236	− 0.00212	− 0.00205	− 0.00205
δ %	0.055	0.0473	0.092	0.0795	0.1	0.1036	0.0979

*C*_*p*_ upper wall							
x/c	0.0997	0.3018	0.5	0.698	0.8044	0.9054	
Exp	0.1327	0.2464	0.1171	0.03457	− 0.01226	− 0.0568	
Two fluid model	0.1394	0.2442	0.1059	0.005576	− 0.0323	− 0.0769	
δ %	0.67	0.22	1.12	2.8994	2.004	2.01	
SST model	0.1416	0.2397	0.101	0.0035	− 0.0345	− 0.0791	
δ %	0.89	0.67	1.61	3.107	2.224	2.23	

*C*_*p*_ lower wall							
x/c	0.1528	0.3018	0.503	0.7512	0.8	0.902	
Exp	0.2955	0.4092	0.384	0.1907	0.1215	0.01449	
Two fluid model	0.346	0.3802	0.349	0.1617	0.1037	− 0.01	
δ %	5.05	2.9	3.5	2.9	1.78	2.449	
SST model	0.357	0.3758	0.351	0.1572	0.1059	− 0.0144	
δ %	6.15	3.34	3.3	3.35	1.56	2.889	

Here δ is the relative deviation.

**Figure 6:**
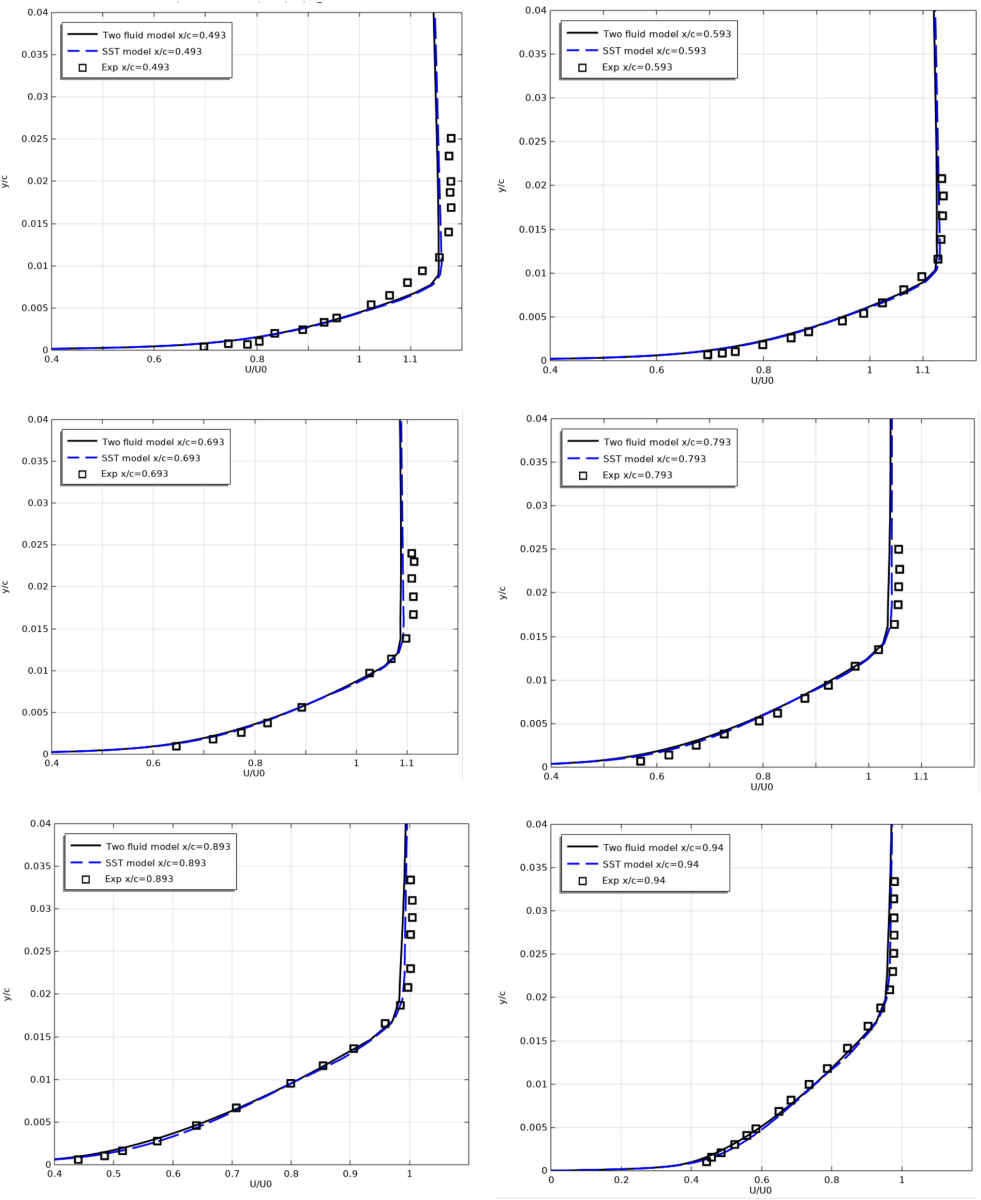

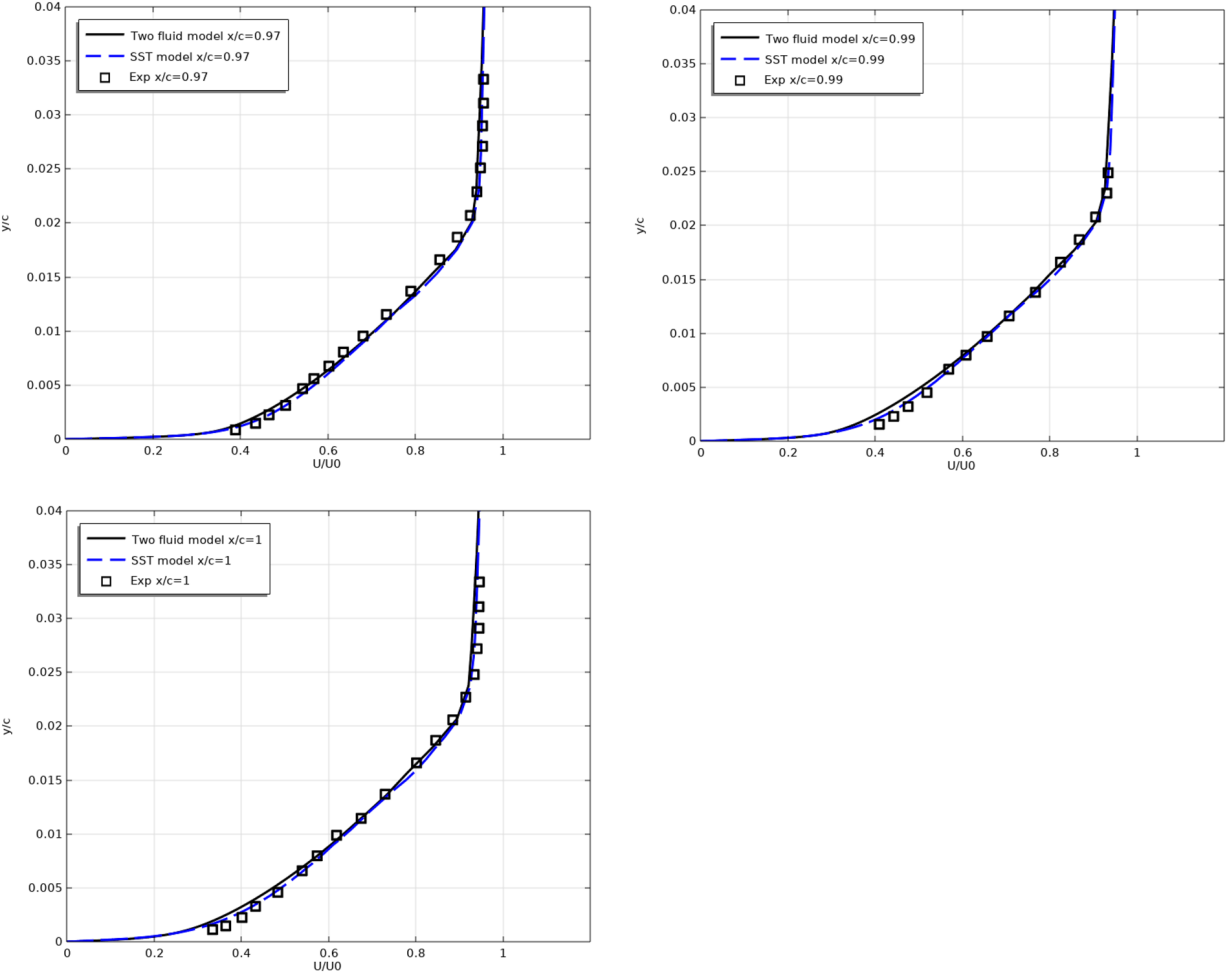
Longitudinal velocity profiles on the top surfaces at x/c = 0.493, 0.593, 0.693, 0.793, 0.893, 0.94, 0.97, 0.99, 1.

**Figure 7:**
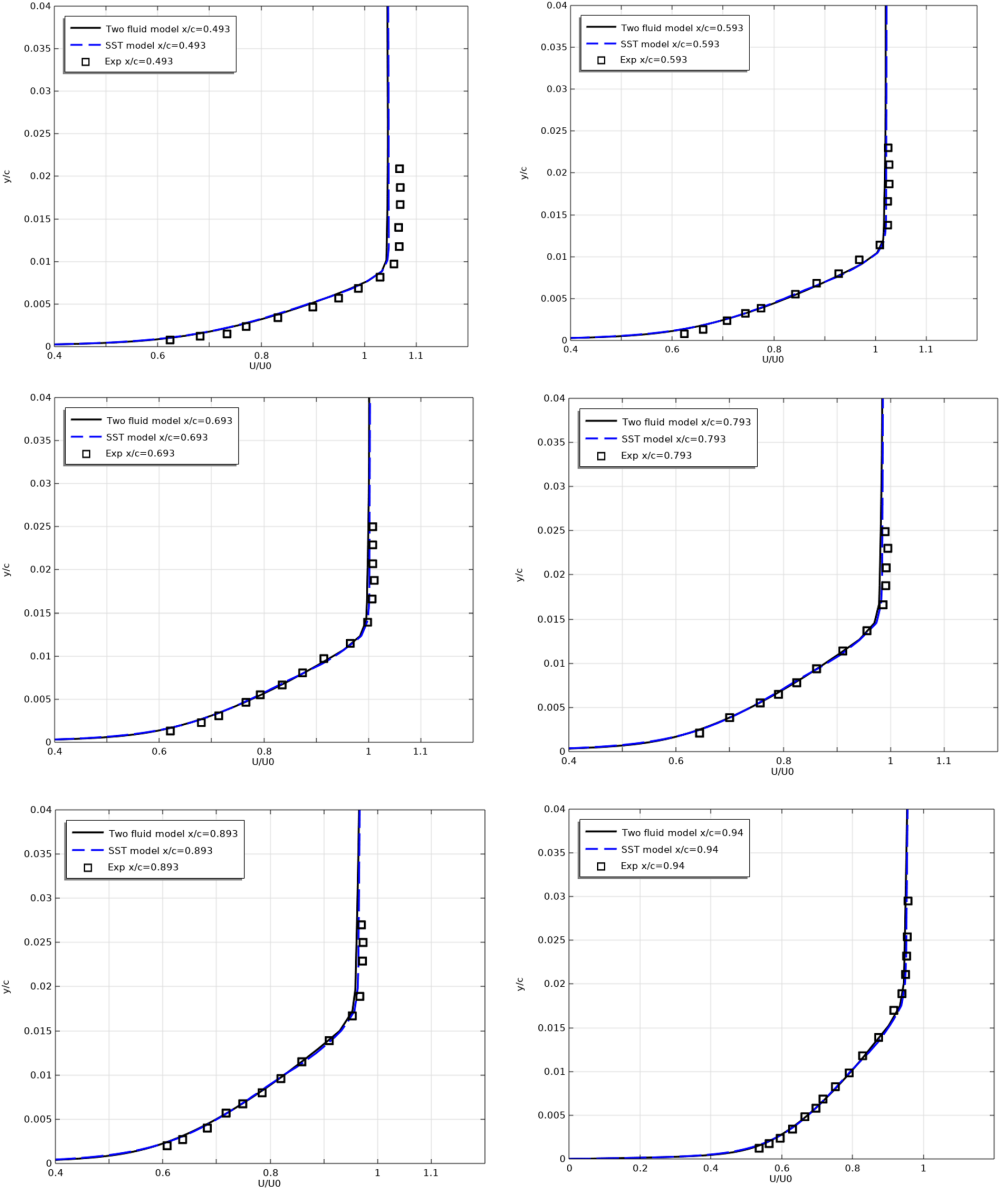

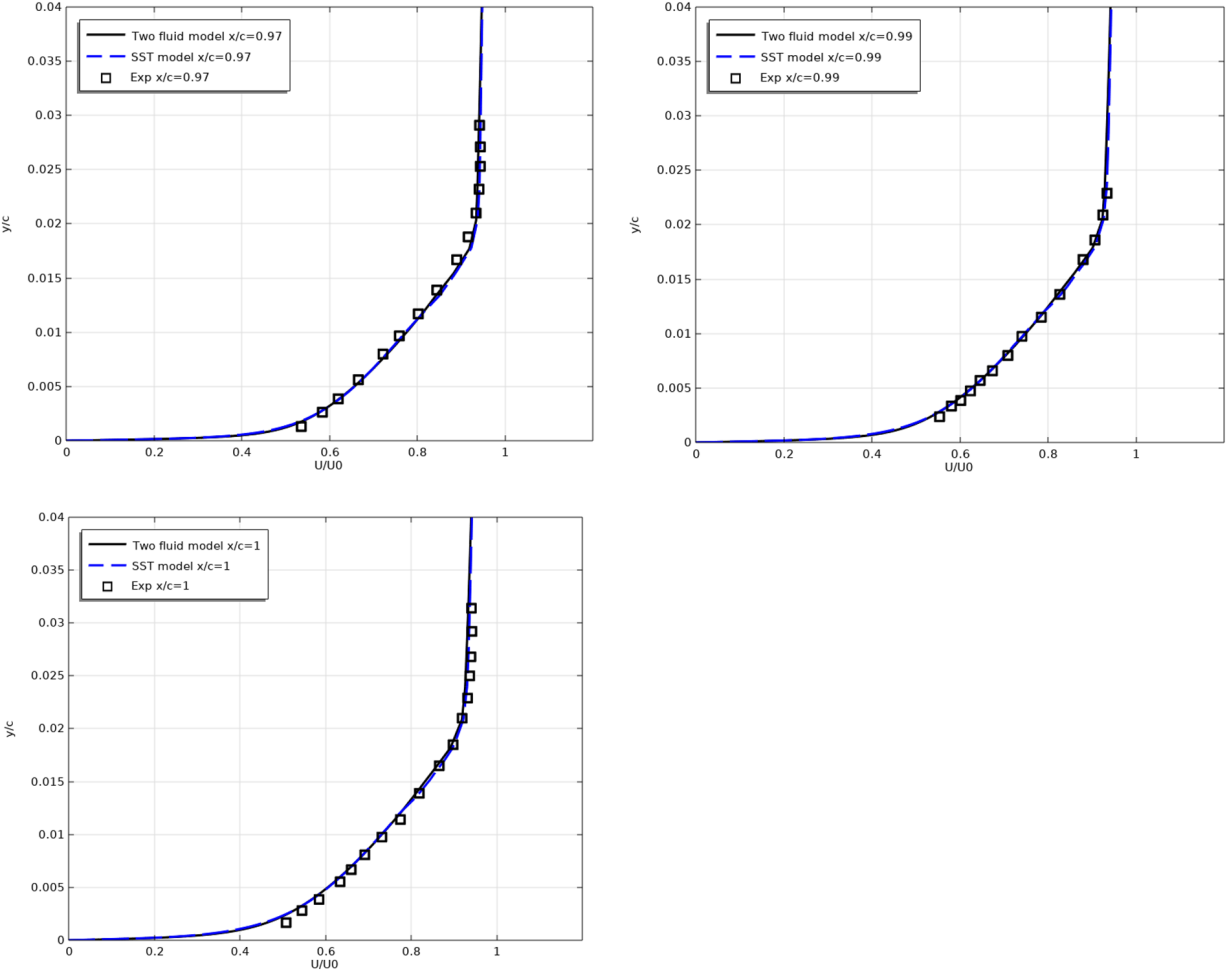
Longitudinal velocity profiles on the bottom surfaces at x/c = 0.493, 0.593, 0.693, 0.793, 0.893, 0.94, 0.97, 0.99, 1.

Figures 6 and 7 show the longitudinal velocity $U/U_{0}$ profiles along the top (Fig. 6) and along the bottom (Fig. 7) surfaces of the profile at different sections along the flow.

As can be seen from Figs. 6 and 7, the results of both models are close to the experimental results.

**Figure 8:**
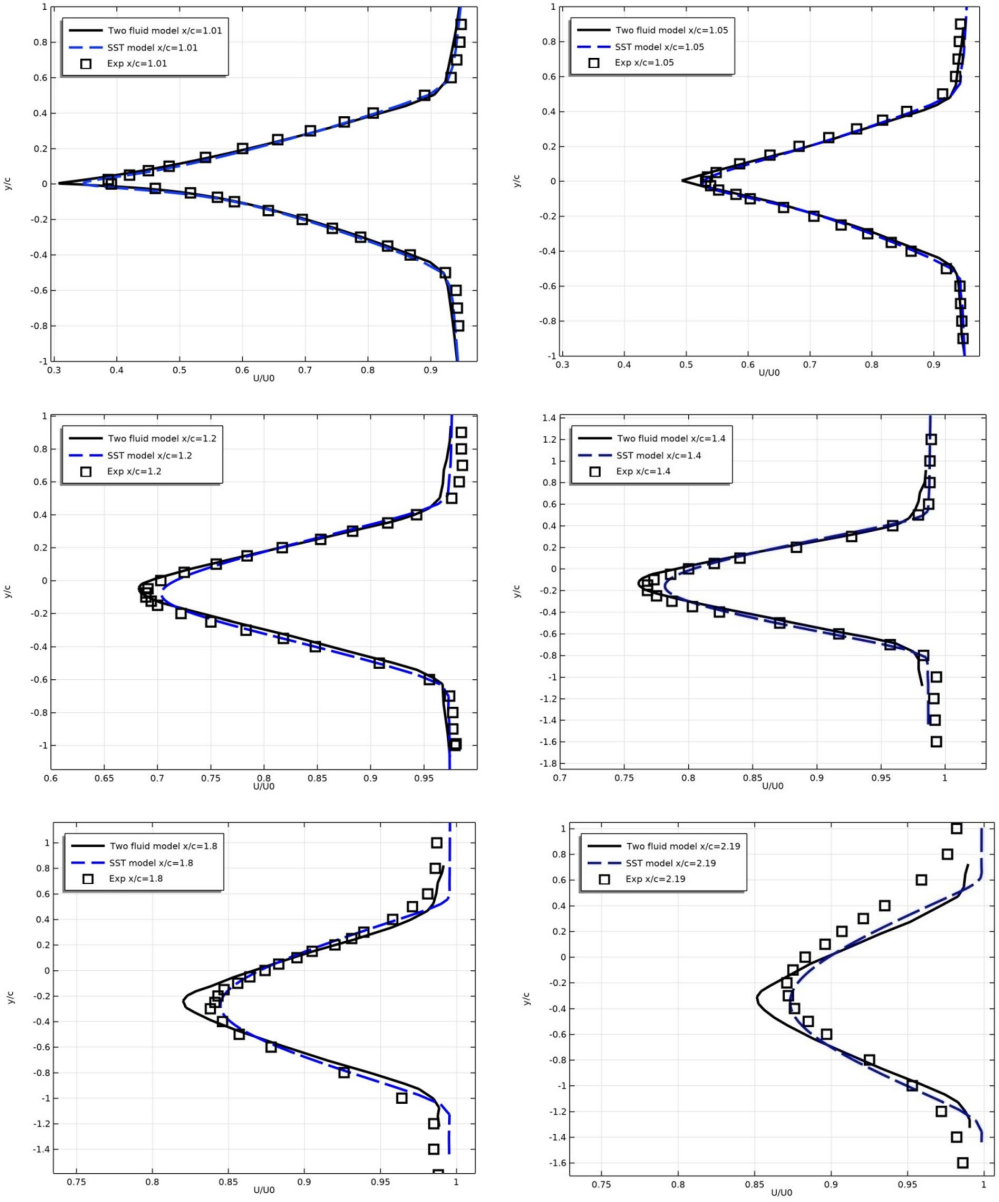
Longitudinal flow velocity in the turbulent wake in sections x/c = 1.01, 1.05, 1.20, 1.40, 1.80 and 2.19.

**Figure 9:**
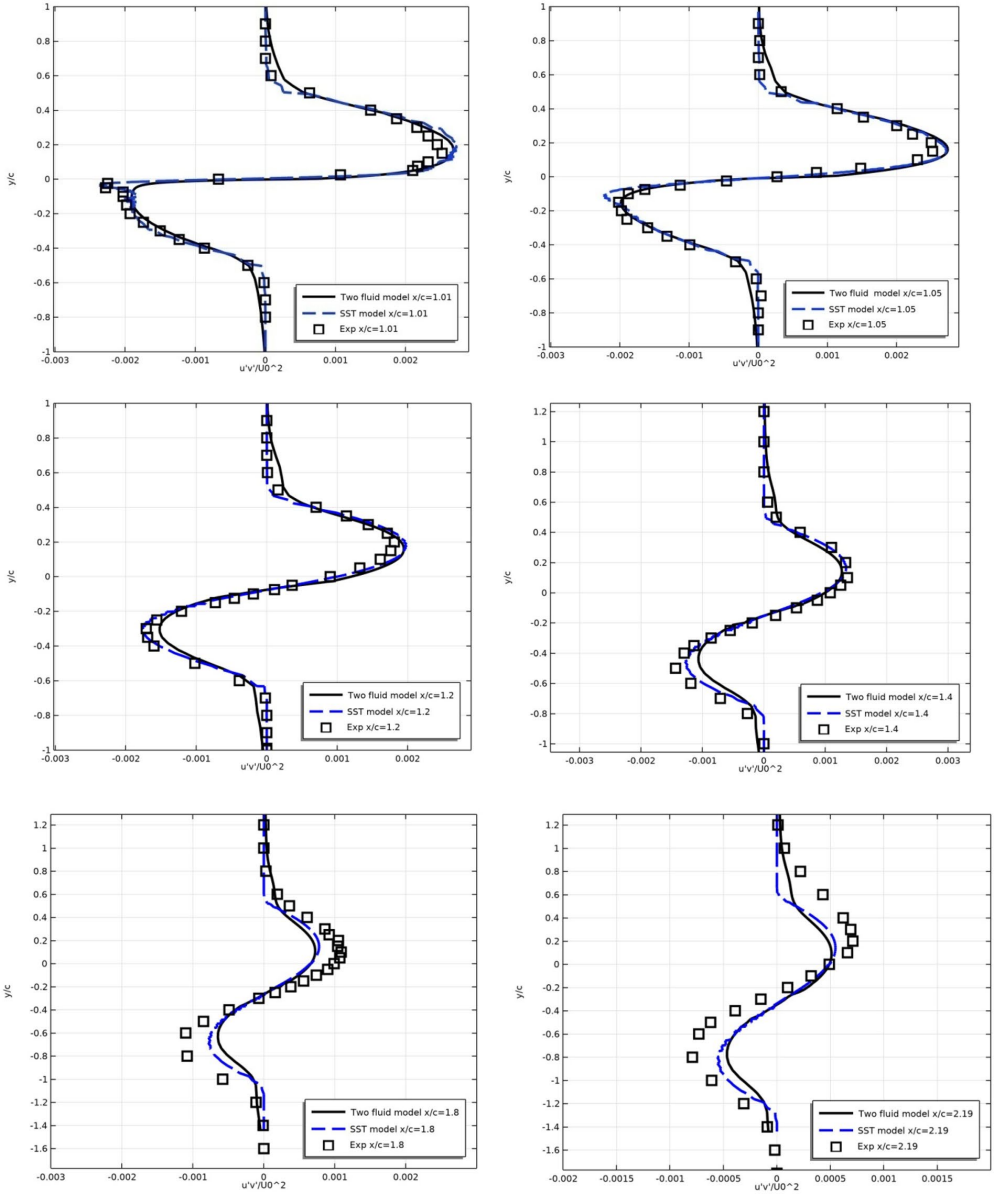
Wake turbulent stresses in sections x/c = 1.01, 1.05, 1.20, 1.40, 1.80 and 2.19.

Below are the numerical results of the models and experiment for the profiles of longitudinal velocity $U/U_{0}$ (Fig. 8) and turbulent stresses $\overline{{u^{\prime}\vartheta^{\prime}}} /U_{0}^{2}$ (Fig. 9) in the wake after the profile at sections x/c = 1.01, 1.05, 1.20, 1.40, 1.80 and 2.19.

Table 2 presents the errors in the deviation of the calculated results from the experimental data in the cross section x/c = 2.19 for *U/U*_*0*_.

**Table 2:** Deviation of tu.

*U/U*_*0*_							
y/c	− 1.19	− 0.797	− 0.498	− 0.29	0.1	0.4	0.6
Exp	0.9717	0.9251	0.8852	0.8714	0.8961	0.935	0.95
Two fluid model	0.9876	0.92	0.8672	0.8524	0.915	0.972	0.98
δ %	1.59	0.51	1.8	1.9	1.89	3.7	3
SST model	0.9865	0.91	0.8785	0.8742	0.9124	0.963	0.99
δ %	1.48	1.51	0.67	0.28	1.63	2.8	4

Here δ is the relative deviation.

Figure 8 shows that the results of both models coincide with each other and with the experimental results for the average computational grid 561 × 97 with high accuracy.

For turbulent stress $\overline{u^{\prime}\vartheta ^{\prime}} /U_{0}^{2}$, there is also a good agreement between the results of models and experiment.

**Figure 10:**
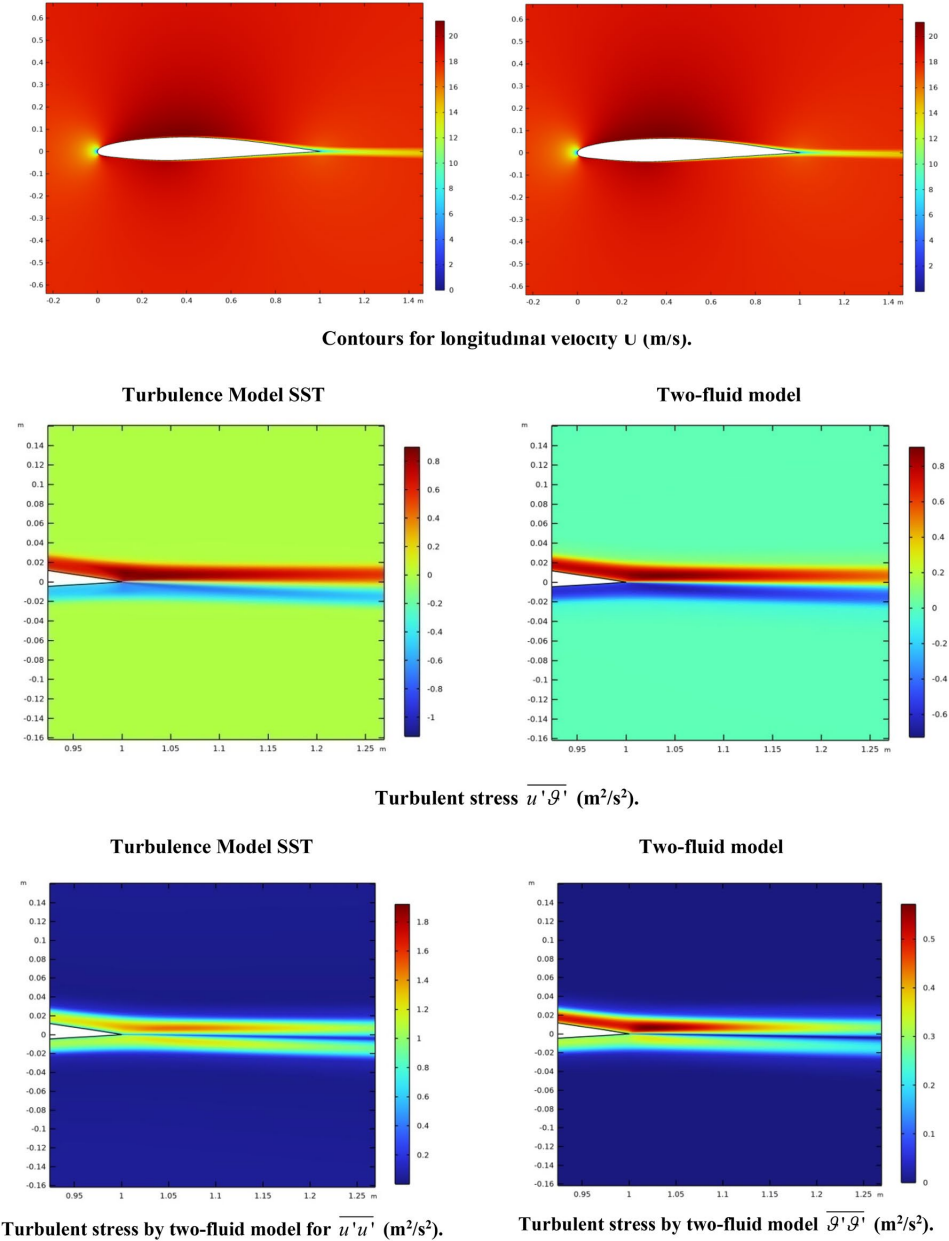
Loops for longitudinal velocity (m/s) and turbulent stresses (m^2^/s^2^).

Figure 10 shows the contours for longitudinal velocity and turbulent stresses.

**Figure 11:**
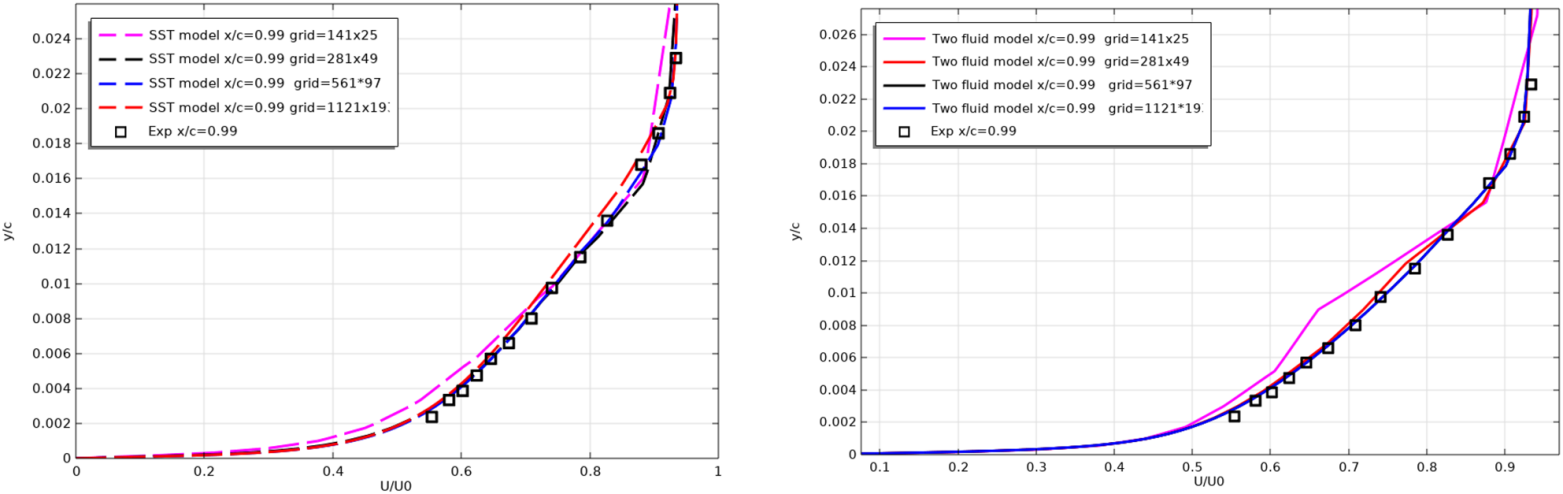
Checking the grid convergence of turbulence models.

To check the grid convergence, Fig. 11 shows the numerical results when the computational grid is changed. The results in section x/c = 0.99 for the bottom surface of the profile are shown.

In Fig. 11 shows that the results of both models do not depend on the resolution of the computational grid.

**Figure 12:**
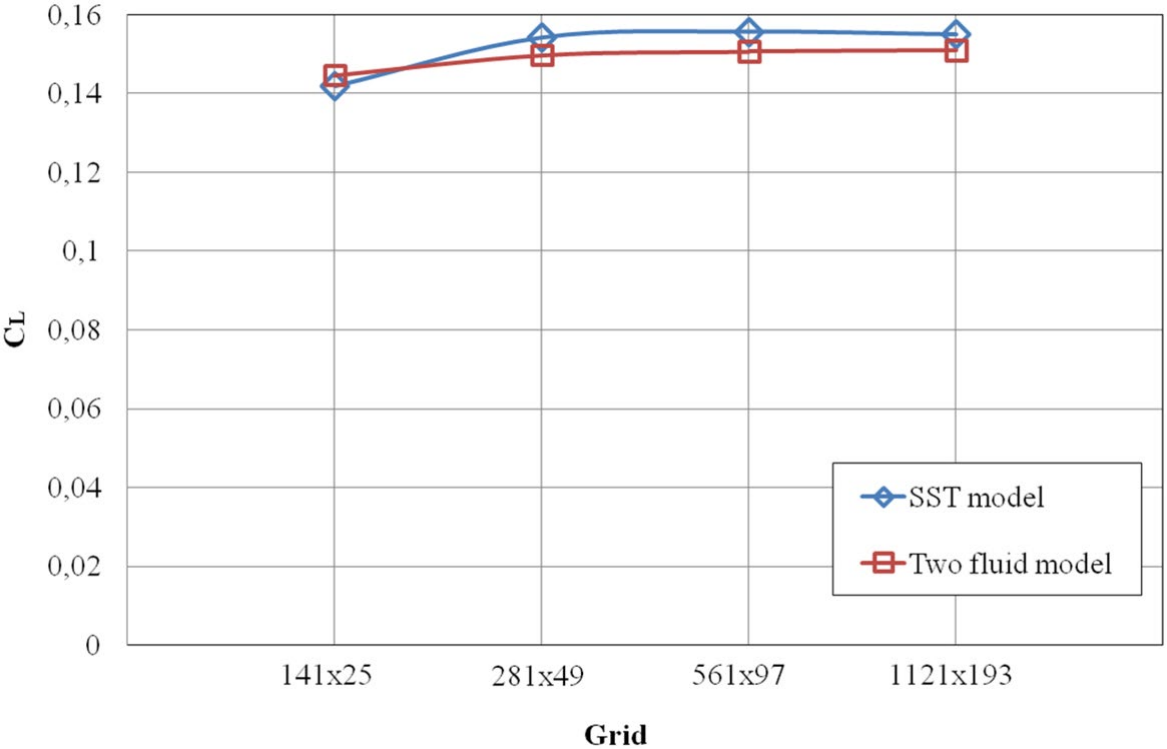
Change in the lift force coefficient C_L_ from the resolution of the computational grid.

Figure 12 shows the change in the lift coefficient C_L_ from the resolution of the computational grid.

It is also clear from Fig. 12 that the results of both models are less sensitive to the resolution of the computational grid.

## Airfoil NACA 4412

**Figure 13:**
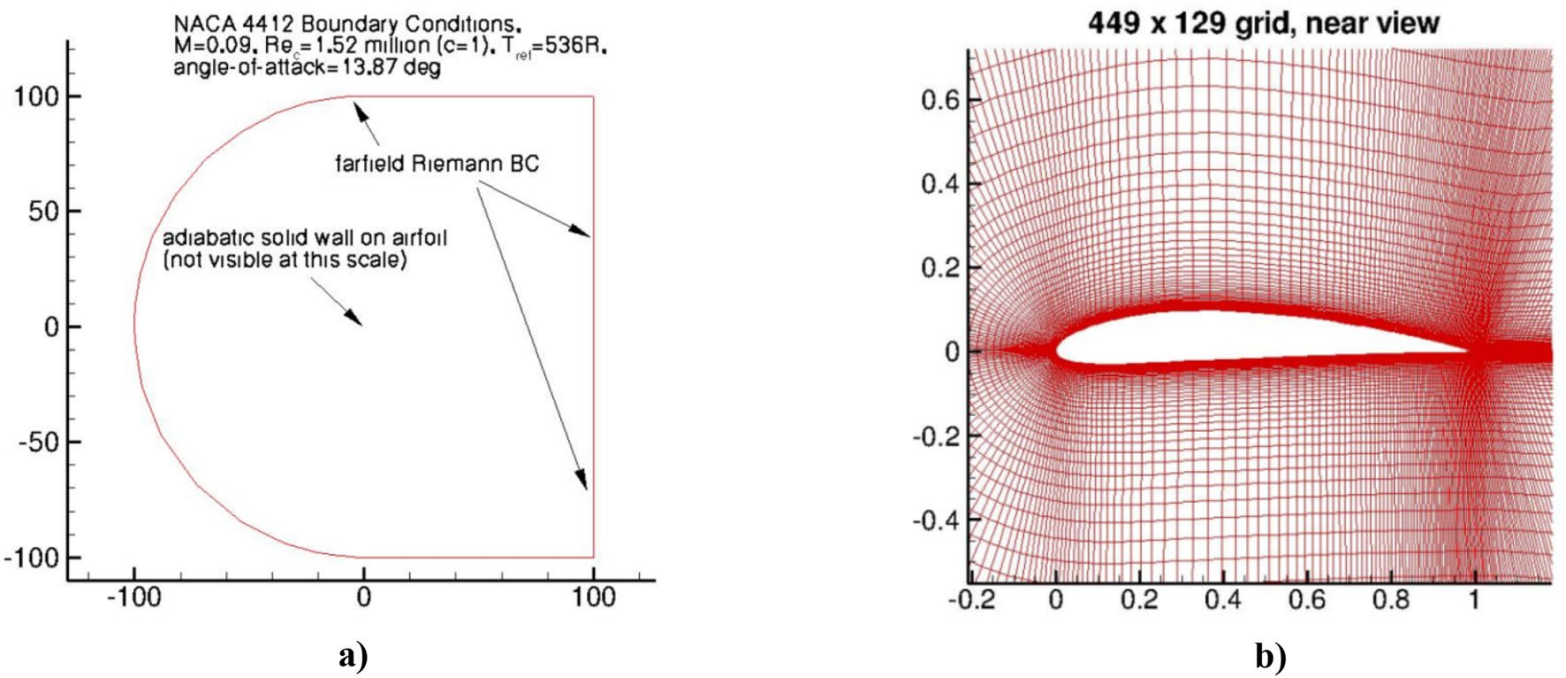
NACA 4412 airfoil. (**a**) Boundary conditions and (**b**) computational grid and domain.

The NACA 4412 airfoil is an airfoil with a maximum thickness of 12% of the chord length, located at 40% of the chord length. It is widely used in various fields, including aircraft wings and propeller blades. This section presents the validation of a two-fluid turbulence model for the NACA 4412 airfoil^43,44^. The case is considered for the Mach number M = 0.09, the angle of attack α = 13.87 ◦ and the Reynolds number Re = 1,520,000. 449 × 129 grid is presented in^7^. The boundary conditions are shown in Fig. 13a, and an illustration of the computational grid and domain is shown in Fig. 13b.

**Figure 14:**
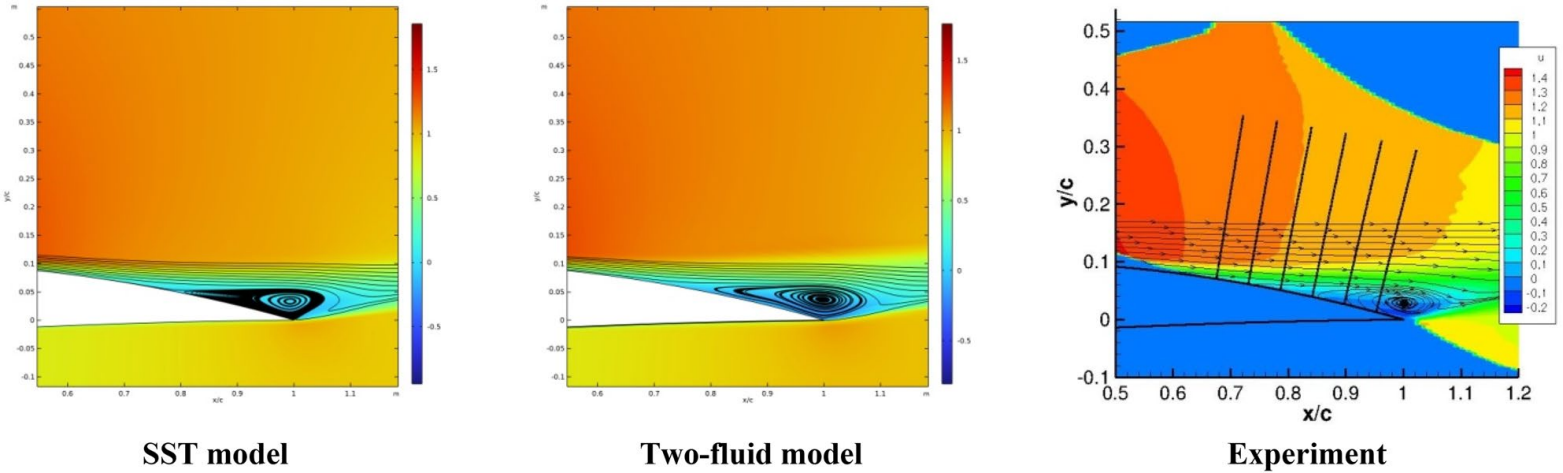
Isolines of flow velocity.

Figure 14 shows the isolines of the flow velocity.

It can be seen from this figure that both models show close results to the experimental data.

**Figure 15:**
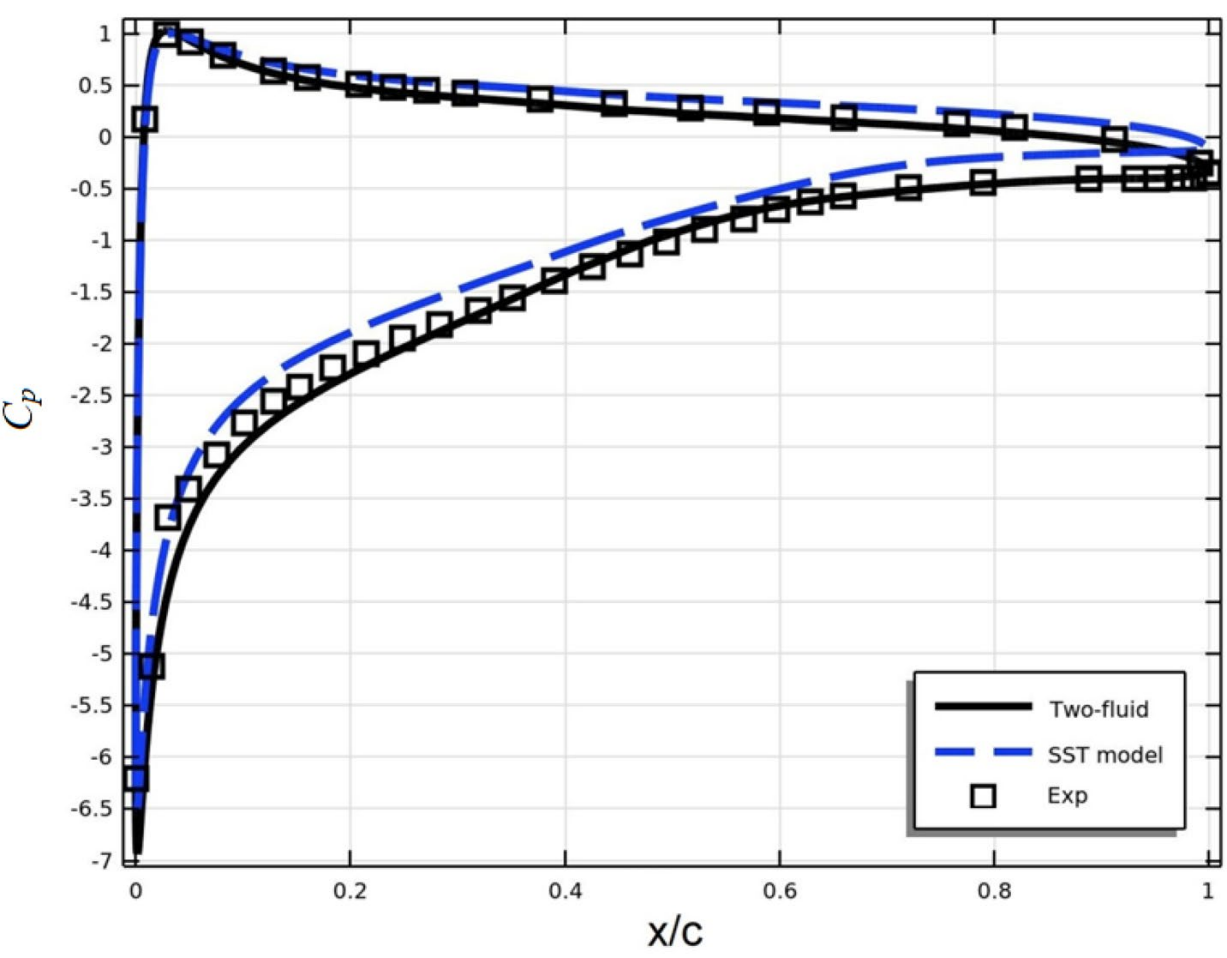
Results for surface pressure coefficient.

Figure 15 shows the distribution of the surface pressure coefficient *C*_*p*_ on the NACA 4412 airfoil. From this figure it can be seen that the results of the two-fluid model are in better agreement with the experimental data. This can be explained by the fact that near the edge of the profile as shown in Fig. 14, a recirculating flow movement occurs, which causes anisotropic turbulence. The SST model uses the Boussinesq hypothesis, which is valid for isotropic turbulence. Therefore, under anisotropic turbulence, the SST results are somewhat worse. As for the two-fluid turbulence model, as shown in previous works, it is capable of describing anisotropic turbulence with great accuracy.

Table 3 presents the deviation of model results from experimental data for *C*_*p*_.

**Table 3:** The relative.

*C*_*p*_ upper wall							
x/c	0.051	0.1827	0.3189	0.5309	0.7185	0.8866	0.951
Exp	− 3.415	− 2.237	− 1.686	− 0.8983	− 0.483	− 0.389	− 0.3983
Two-fluid model	− 3.677	− 2.372	− 1.7203	− 0.822	− 0.5	− 0.4152	− 0.39831
δ	0.262	0.135	0.0343	0.0763	0.017	0.0262	0.000005
SST model	− 3.1694	− 1.974	− 1.3893	− 0.6525	− 0.2711	− 0.1335	− 0.144
δ	− 0.2456	− 0.263	− 0.2967	− 0.2458	− 0.2119	− 0.2555	− 0.2543

*C*_*p*_ lower wall							
x/c	0.079	0.2088	0.3752	0.5872	0.7642	0.9135	0.9926
Exp	0.8135	0.5169	0.3898	0.2372	0.1525	− 0.0169	− 0.2203
Two-fluid model	0.7711	0.5084	0.338983	0.177	0.08447	− 0.0338	− 0.2288
δ	0.0424	0.0085	0.0508	0.0602	0.0680	0.0169	0.0085
SST model	0.8474	0.5762	0.483	0.3644	0.2627	0.1355	− 0.0254
δ	− 0.0339	− 0.0593	− 0.0932	− 0.1272	− 0.110	− 0.152	− 0.1949

Hear $$\delta ={C}_{p.exp}-{C}_{p.model}$$.

**Figure 16:**
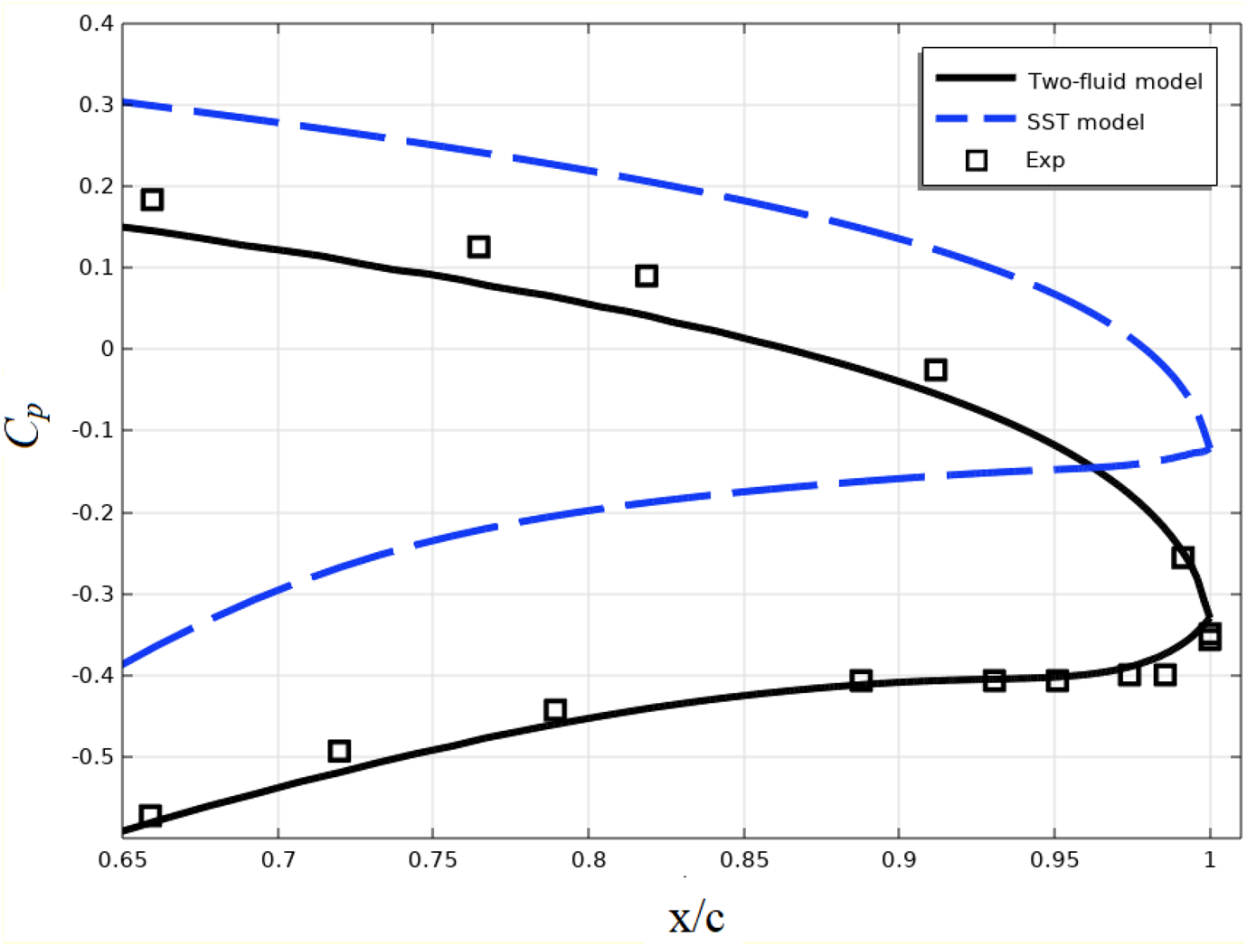
Pressure coefficient distribution at trailing of the edge.

The controls of the airliners are mainly located on the edge of the profile. Therefore, the accuracy of calculating the distribution of the pressure coefficient near the profile edge is of great practical importance. In this regard, Fig. 16 shows the distribution of the pressure coefficient at trailing of the edge. From this figure it can be seen that the results of the two-fluid model are significantly better than the results of the SST model.

**Figure 17:**
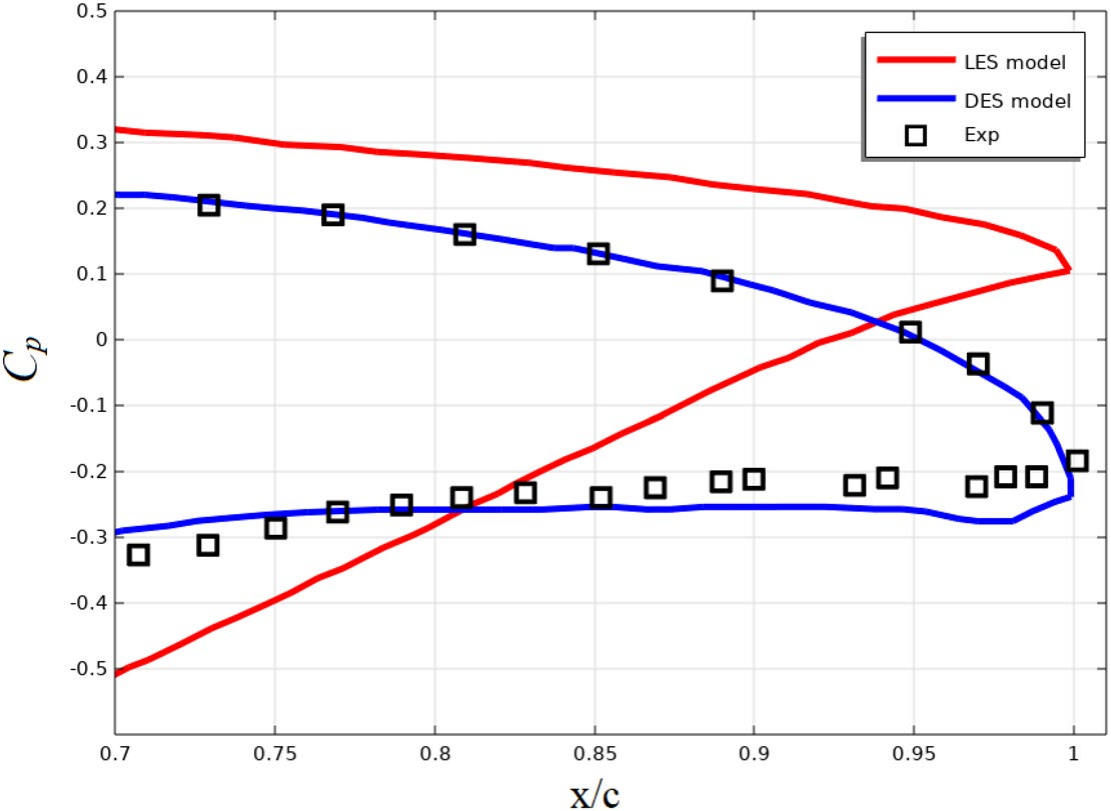
Pressure coefficient distribution near the profile edge.

Figure 17 shows the same results using different models for the NACA4412 airfoil at an angle of attack of 12^0^ and Reynolds number Re = 1.64·10^6^. These results are obtained from the work^45^.

The results presented in Figs. 16 and 17 show that the accuracy of the two-fluid model is higher than that of the RANS models and no worse than that of the LES and DES models.

**Figure 18:**
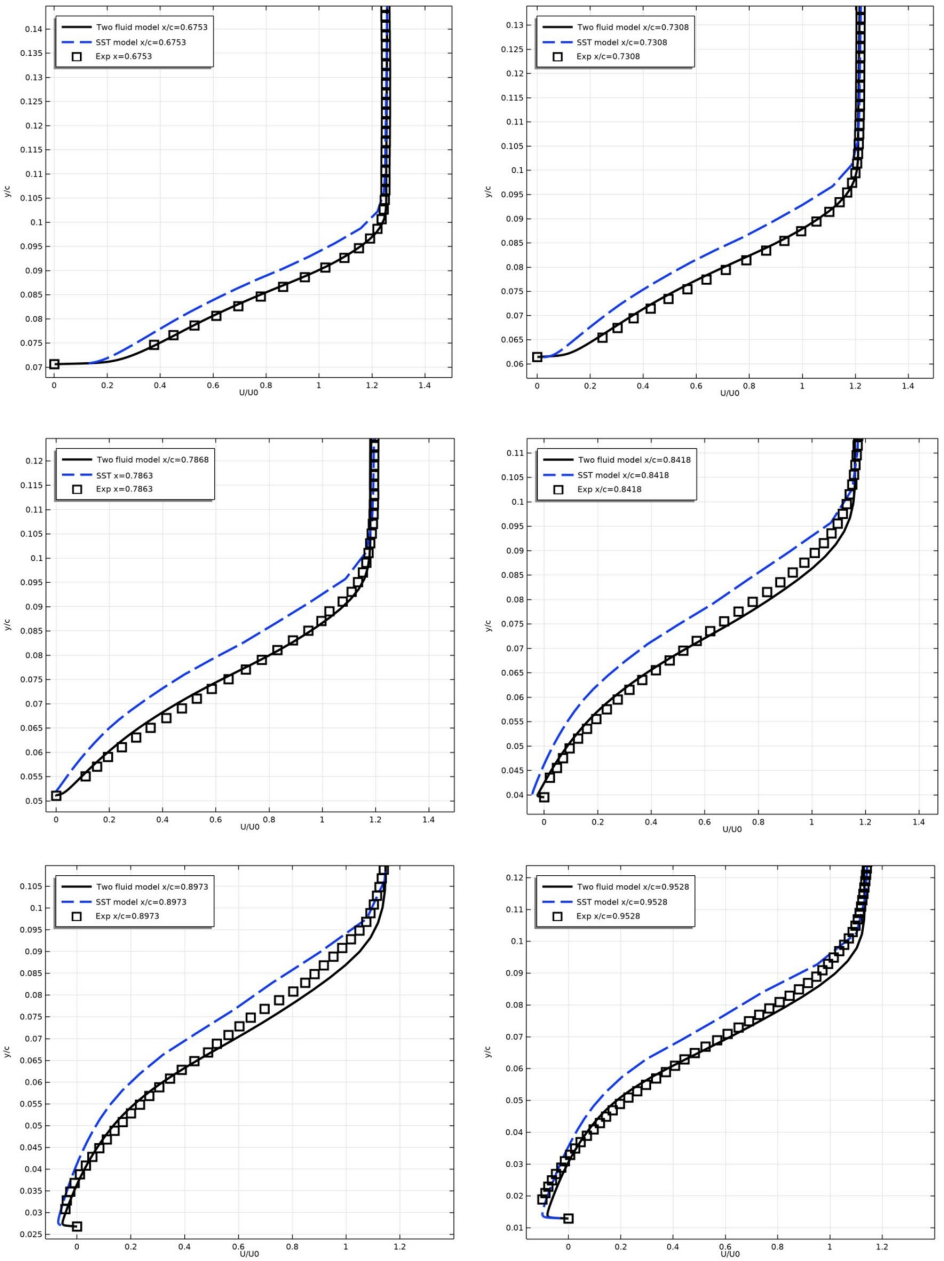
Longitudinal velocity profiles on the upper surface of the profile at x/c = 0.6753, 0.7308, 0.7863, 0.8418, 0.8973, 0.9528.

Figure 18 shows the longitudinal velocity $U/U_{0}$ profiles along the upper surface of the NACA 4412 airfoil at different sections downstream.

Here, too, there is a correspondence between the results of the models and experimental measurements. It can be seen from the figure that the velocity profiles according to the results of the SST model slightly deviate from the experiment in all sections, and the results of the two-fluid model are in good agreement with the experimental data.

Table 4 presents the relative deviations of the model results from the experimental data in the cross section x/c = 0.6753 for U/U_0_.

**Table 4:** The Cp model.

U/U_0_							
y/c	0.074	0.08	0.086	0.09	0.094	0.098	0.102
Exp	0.3785	0.6173	0.873	1.032	1.153	1.22	1.24
Two fluid model	0.3831	0.6037	0.859	1.025	1.15	1.222	1.25
δ %	0.46	1.36	1.4	0.7	0.3	0.2	1
SST model	0.3014	0.4948	0.7127	0.88	1.03	1.157	1.22
δ %	7.71	12.25	16.03	15.2	12.3	6.3	2

Here δ is the relative deviation.

**Figure 19:**
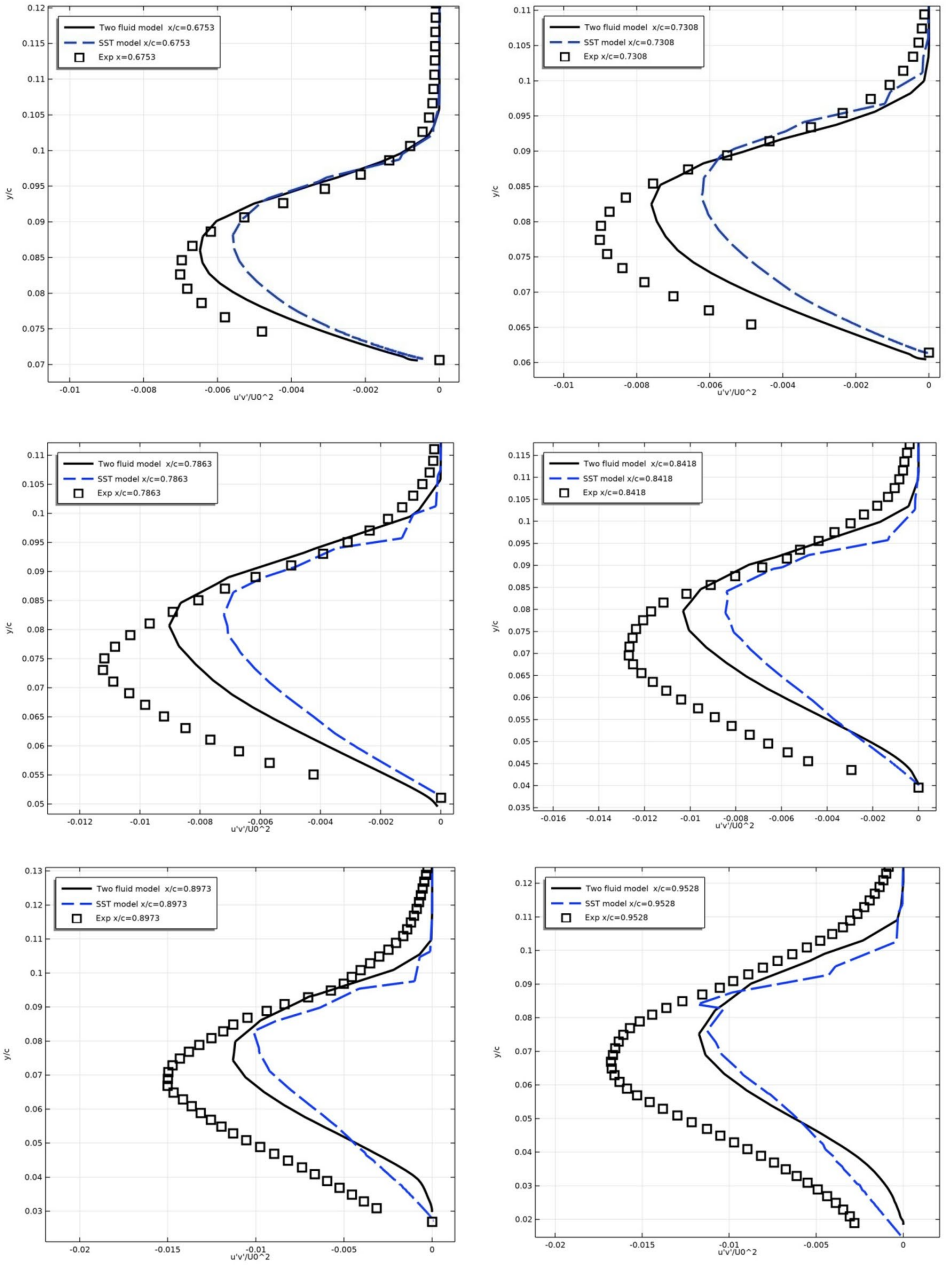
Profiles of turbulent stresses on the upper surface in sections x/c = 0.6753, 0.7308, 0.7863, 0.8418, 0.8973, 0.9528.

Figure 19 shows the turbulent stress $\overline{{u^{\prime}\vartheta^{\prime}}} /U_{0}^{2}$ along the top surface for the NACA 4412 airfoil in different sections.

From Fig. 19 it is clear that the turbulent stresses correspond to the experimental data worse than the averaged values. This is due to the fact that they have small values and to improve compliance it is necessary to use calculation schemes of a higher order of accuracy.

Calculation time for problem NACA 4412: 420 iterations and 1740s in the SST model, and 380 iterations and 1588 s in the two-fluid model. All calculations were performed on a computer with a 2.5 GHz quad-core Intel i5-7300HQ processor, 16 GB DDR3 RAM, a 1024 GB hard drive, and Windows 7 (64-bit).

## Conclusion

This article demonstrates the capability of the two-fluid turbulence model in the Comsol Multiphysics software package, which uses the finite element method. To stabilize the model equations, a stabilizer based on the Galerkin least squares method was used. For validation of the model and verification of the numerical algorithm, the problems of flow past a flat plate with a zero pressure gradient, as well as DSMA661 and NACA 4412 airfoils with angles of attack of 0 and 13.87 degrees, respectively, are considered. For the first time in the Comsol Multiphysics program, a two-fluid model was introduced and the results were obtained. To verify the created program, the results obtained are compared with the results of other models, as well as with experimental data. It is shown that the results of the two-fluid model correspond better to experimental data than the results of other RANS models and are no worse than the results of the LES and DES models. In addition, it is shown that the two-fluid model requires less computational resources than the SST model. Therefore, the two-fluid model can be recommended for calculating engineering problems of turbulent hydrodynamics.

## Data Availability

The datasets generated and/or analysed during the current study are available in the [Murodil Madaliev] repository Results.zip, https://drive.google.com/file/d/1aZsndL533a10yrLGX522Asx0SYVMP8xG/view?usp=drive_link. The datasets used and/or analysed during the current study available from the NACA TMR web site https://turbmodels.larc.nasa.gov/flatplate_val.html for the first chapter, https://turbmodels.larc.nasa.gov/airfoilwake_val.html for the second chapter, https://turbmodels.larc.nasa.gov/naca4412sep_val.html for the third chapter.

## Abbreviations

*V*_*i*_: Component of the average flow velocity
*ϑ*_*i*_: Component of the relative velocity
*ν*_*ji*_: Effective kinematic viscosity tensor arising from the relative motion of fluids
*p*: Pressure in fluid mixture
*K*_*f*_: Scalar function of the friction force
*C*_*s*_: Shear coefficient
*ν*: Molecular viscosity
t: Time
C_1_: First constant in the coefficient of friction
C_2_: Second constant in the coefficient of friction
*λ*_max_: Largest root of the characteristic equation
*S*_*ij*_: Strain rate tensor of a mixture of fluids
Re: Reynolds number
*d*: Nearest distance from a given point to a solid wall
*ρ*: Density
*C*_*p*_: Pressure coefficient
RANS: Reynolds-averaged Navier–Stokes
SST: Shear stress transport
SA: Spalart–Allmaras
DES: Detached Eddy simulation
LES: Large eddy simulation
DNS: Direct numerical simulation
FEM: Finite element method
SUPG: Streamline-upwind/Petrov–Galerkin
PDE: Partial differential equations
CFD: Computational fluid dynamics
GLS: Galerkin least squares
PARDISO: Parallel direct sparse solver for clusters
